# Targeting vascular inflammation and endothelial dysfunction with nanobodies: emerging therapeutic strategies for cardiovascular disease

**DOI:** 10.3389/fimmu.2026.1861674

**Published:** 2026-09-15

**Authors:** Ibrahim Aljaezi

**Affiliations:** 1 Department of Pharmacology, College of Pharmacy, Najran University, Najran, Saudi Arabia; 2 Health Research Center, Najran University, Najran, Saudi Arabia

**Keywords:** cardiovascular diseases, endothelial activation, nanobodies, therapeutic strategies, vascular inflammation

## Abstract

Despite a myriad of medicinal breakthroughs in lipid-lowering and antithrombotic therapies, cardiovascular diseases (CVDs) are still the main killers worldwide. According to the consolidated scientific evidence, atherosclerosis, ischemic heart disease, and other vascular pathologies are attributed to endothelial dysfunction and vascular inflammation. The standard treatments’ failure to adequately control the inflammatory and endothelial pathways has led researchers to biologics that are molecularly specific. Nanobodies (Nbs) are single-domain antibody fragments obtained from camelid heavy-chain antibodies. Due to their small size, good tissue penetration, and stability, they are regarded as therapeutic agents and diagnostic tools. This paper is focused on the current and potential future uses of nanobodies in the treatment of endothelial activation, the blockade of adhesion molecule expression, and the management of cytokine-induced vascular inflammation. The literature shows that diagnostic nanobody tracers achieve high accuracy for vascular inflammation imaging, yet therapeutic Nbs exist only in preclinical stages. PANX1-blocking Nbs show promise for treating ischemia–reperfusion injury, and IL-1β-targeted Nbs demonstrate effectiveness in reducing hypoxia-induced damage to endothelial cells. The therapeutic potential of Nbs has been proven by caplacizumab, which functions as an anti-vWF nanobody, but its approved medical use exists only for hematologic disorders. The therapeutic potential of Nbs remains unexplored for ROS, ox-LDL, AT1R, and chemokines CCL2 and CCL5 because researchers have not developed corresponding nanobody-based interventions. The review demonstrates how nanobody engineering advances combined with current molecular knowledge create a promising yet underdeveloped therapeutic area for cardiovascular medicine, which could use precise biologic treatments to restore vascular equilibrium and fight inflammation, thus transforming future CVD treatment approaches.

## 1 Introduction

Cardiovascular diseases (CVDs) are the major causes of death worldwide. These are diseases that could potentially lead to heart failure, atherosclerosis, coronary heart disease, myocardial infarction, and the like. As per the World Heart Federation survey report, CVDs are responsible for 20.5 million deaths globally every year (1). Nevertheless, since most of the CVD cases and resultant deaths are primarily due to risk factors that can be changed, and some of these factors are global and others vary with the economic conditions of the region, the death rates associated with CVDs are expected to rise over the next decade. This is because risk factors, including obesity, elevated cholesterol, hypertension, diabetes, smoking, physical inactivity, poor nutrition, and an aging population, are anticipated to increase significantly (2). This calls for targeted health policies. According to estimates, cardiovascular diseases were responsible for 17.7 million of the 55 million deaths that occurred globally in 2017 (3). Sadly, estimates suggest that by 2030, the number of deaths from CVD will rise to over 23.6 million (4).

Many people consider atherosclerosis (AS) to be the pathological foundation of CVD. It is an inflammatory disease that is systemic, chronic, and progressive (5). Accumulation of lipids and fibrous materials in the vascular endothelium causes decreased arterial elasticity, arterial wall thickening, and vascular lumen narrowing, leading to the disease. Thrombosis brought about by the progressive breakdown of atherosclerotic plaques produces dangerous cardiovascular emergencies (6). The vascular endothelium plays an important role in the maintenance of cardiovascular homeostasis. The vascular endothelium, while maintaining a protective barrier between blood and the vessel walls, regulates blood vessel tone, permeability, and metabolic processes (7). Research shows that endothelial cell dysfunction (ED) represents the initial critical step that leads to atherosclerosis development (8). The combination of aging, autoimmune diseases, psychological stress, microbial and viral infections, and traditional cardiovascular risk factors leads to endothelial cell damage. ED is not always irreversible, which is important to note. Early-stage damaged endothelial cells may return to normal function when pathological triggers are eliminated, suggesting a possible window for treatment (9).

A balanced diet, regular exercise, and quitting smoking are all important components of healthy lifestyle interventions that help improve ED and slow the progression of AS (10). Though they primarily slow rather than reverse AS, diets such as the Mediterranean pattern increase antioxidant and anti-inflammatory capacity, and excessive restriction or imbalance may affect nutrient availability (11). Even though exposure to long-duration, highly intense exercise can worsen endothelial damage by increasing oxidative stress and inflammation, moderate-intensity exercise maximizes endothelial signaling, increases NO, and decreases ROS (12). In the same manner, quitting smoking reduces the burden of ROS and vascular aging (13), although the benefits are gradual and cannot completely undo previous endothelial damage. Drug therapies that target important vascular pathways are one type of additional treatment for ED. For example, while statins primarily slow AS and may cause myopathy or liver dysfunction, they also improve endothelial function by lowering LDL levels and increasing eNOS activity and NO release (14). Although antihypertensive medications such as calcium channel blockers and RAS inhibitors lessen inflammation and oxidative stress, their advantage is in halting additional damage rather than fixing dysfunction; they may cause adverse effects like edema and electrolyte imbalance (15). Similar to this, antidiabetic medications such as metformin, SGLT2 inhibitors, and GLP-1 agonists enhance endothelial NO production and lower ROS, but they primarily slow the progression of vascular injury and may result in metabolic or gastrointestinal issues (16). Finally, antiplatelet medications such as clopidogrel and aspirin improve endothelial protection by reducing platelet aggregation and inflammation, but they also increase the risk of bleeding and do not reverse dysfunction (17).

By increasing medication bioavailability, boosting site-specific accumulation, and lowering systemic toxicity, nanoparticle-based drug delivery systems have become viable platforms for the targeted treatment of cardiovascular disorders. In Apoe^−/−^ mouse models of atherosclerosis, statins like atorvastatin have been added to hyaluronan-coated and macrophage membrane-coated nanoparticles (HA-ATV-NP and Oxi-COS/MM-AT-NPs) to significantly suppress vascular inflammation and enhance plaque stabilization (18). Similarly, through targeted delivery to atherosclerotic lesions, rapamycin-loaded nanoparticles, such as leukocyte membrane-coated liposomes and magnetic nanoparticles functionalized with profilin-1 antibodies, have shown anti-inflammatory benefits (19). In addition to conventional medications, nanoparticle platforms have been used to improve targeted accumulation after myocardial infarction by delivering traditional Chinese medicine components, such as saponins and salvianolic acid B, to ischemic myocardium via RGD peptide-modified liposomes. Furthermore, nucleic acid-based nanocarriers carrying siRNA or microRNA have demonstrated potential to enhance efferocytosis, restore vascular smooth muscle cell homeostasis, and lessen restenosis (20), while high-density lipoprotein (HDL)-mimetic nanoparticles have been investigated for the delivery of small-molecule agonists and inhibitors to reduce monocyte recruitment and plaque inflammation. When taken as a whole, these nanoplatforms offer several benefits over traditional drug delivery, including improved targeting effectiveness, regulated drug release, extended therapeutic efficacy, and fewer side effects (21). Although more clinical validation is necessary, their adaptability makes them appealing candidates for precision medicine methods in cardiovascular disease since it allows the delivery of a variety of therapeutic cargoes.

Despite their advantages, the poor solubility, non-specific cytotoxicity, first-pass metabolism, absorption, low bioavailability, and poor biocompatibility of current cardiovascular medications have forced scientists to look for other treatment methods or to improve the current ones. Significant advancements have been made in the treatment of atherosclerosis in recent years, especially in the areas of vascular inflammation and ED mitigation. In addition to these treatments, new strategies like genetic interventions and stem cell therapies are being developed. Despite these developments, the field still faces difficulties in guaranteeing safety, large-scale production, cost, availability, and long-term efficacy (22).

The dual roles of antibodies, especially, have garnered attention because they can both neutralize other antigens and modified lipoproteins and form immune complexes that worsen vascular injury. Antibody-based strategies in CVDs have been validated by therapeutic mAbs targeting pathways such as PCSK9 or pro-inflammatory cytokines. Their long existence duration creates a challenge for medical imaging procedures: their large size prevents them from being internalized into atherosclerotic plaque tissue, and their Fc region promotes inflammatory responses via the activation of effector cells. The limitations that traditional antibodies currently present in vascular targeting reflect a gap between targets they could potentially interfere with therapeutically and targets they actually reach and interact with (22). To overcome current obstacles, nanobodies (Nbs) have evolved as single-domain fragments and developed into good tools. The compact dimensions of Nbs allow them to reach deeper levels within vascular tissue and access endothelial cells and intraplaque targets inaccessible to IgGs. The development of Nbs enables researchers to create therapeutic and diagnostic tools that unite precise medical treatments with vascular biological understanding. The field of CVD research remains in its initial stages even though researchers have established robust evidence. The majority of reviews about Nbs focus on their use for infectious disease treatment and cancer therapy instead of cardiovascular inflammation and endothelial repair. We aim to establish a vital connection between nanobody-based treatments and cardiovascular disease mechanisms through diagnostic and therapeutic approaches that focus on this specific area.

## 2 Methodology

To find relevant studies on Nbs and their developing uses in cardiovascular disorders, including endothelial dysfunction and vascular inflammation, a thorough literature search was conducted utilizing the PubMed, Scopus, Google Scholar, and Web of Science databases. Combinations of keywords such as “nanobody,” “single-domain antibody,” “VHH,” “endothelial dysfunction,” “vascular inflammation,” “atherosclerosis,” “cardiovascular disease,” “targeted therapy,” “molecular imaging,” and “therapeutic applications” were used in the search. The majority of the research that was published between 2010 and 2025 was taken into account, but when appropriate, previous seminal papers that described the discovery of Nbs, their structural characteristics, and basic engineering techniques were also included.

The chosen literature comprised original research articles and review papers that addressed the structure, generation, target recognition, therapeutic design, imaging applications, distribution methods, and prospective function of Nbs in the management of cardiovascular disease. Priority was given to studies explaining how Nbs control inflammatory pathways, atherosclerosis, endothelial dysfunction, and other cardiovascular diseases. Since the use of Nbs in cardiovascular applications is still in its infancy, pertinent research from other therapeutic domains was also taken into account when discussing transferable tactics, such as pharmacokinetic optimization, half-life extension techniques, and targeted delivery platforms. Articles that have nothing to do with cardiovascular applications or nanobody technology were not included. In order to highlight present developments, constraints, and prospects for nanobody-based therapies in cardiovascular disorders, the gathered data were rigorously examined.

## 3 Nanobody technology: design and engineering

### 3.1 What are nanobodies?

Heavy-chain-only antibodies (HcAbs) naturally occur in camelids, nurse sharks, and spotted ratfish (**Figure 1**). Interestingly, it was found that HcAbs are the evolved version of conventional antibodies (cAbs) in camelids, due to which it was suggested that they possess particular functional characteristics that cAbs do not (23). This may be due to differences in structure and size. The most common human serum antibody isotype is human immunoglobulin G2. IgG consists of two light chains, each with one constant CL and flexible VL domain, and two heavy chains, each with three constant CH1–3 and a flexible VH domain. HcAbs, in turn, contain only two heavy chains, specifically one variable heavy-chain VHH domain and two constant heavy chains. Therefore, HcAb establishes antigens through the VHH domain (24).

**Figure 1:**
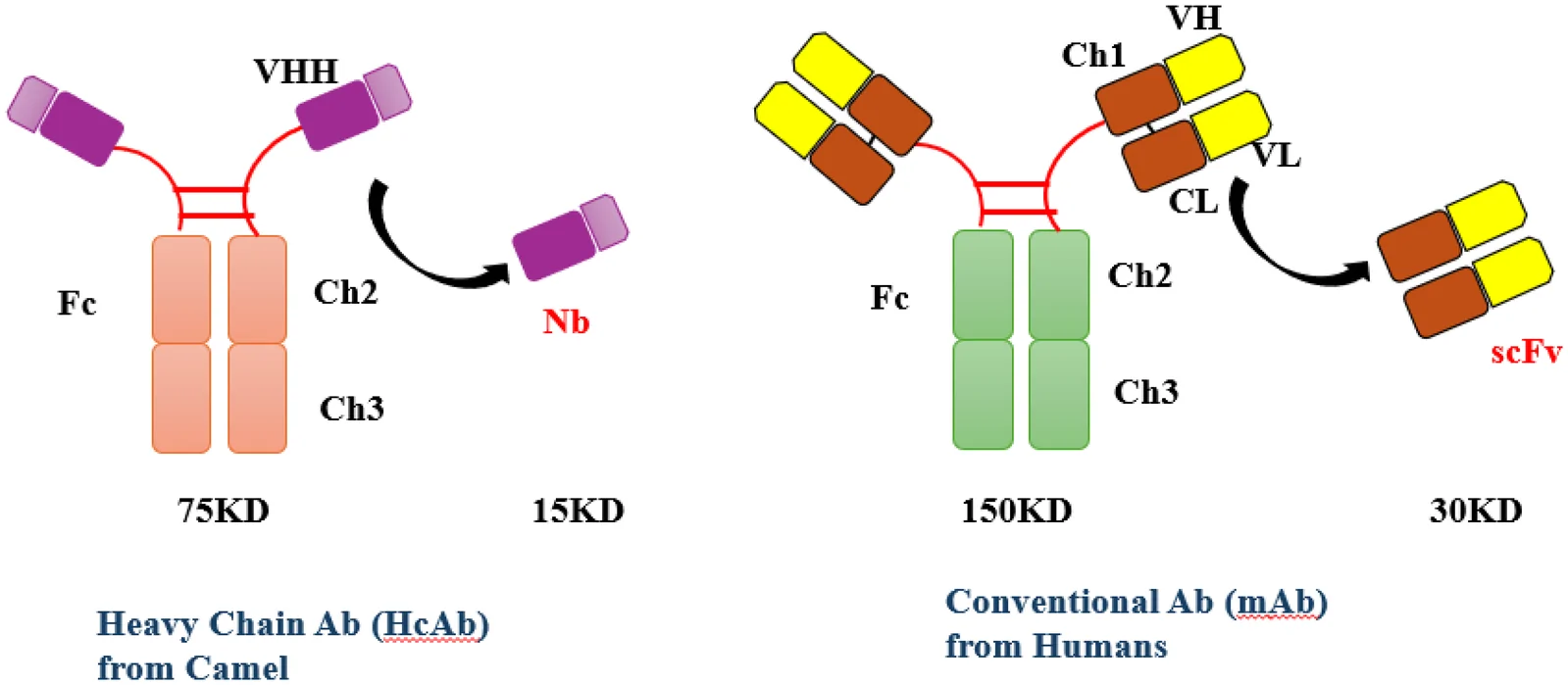
Comparison of nanobody (Nbs) with conventional antibody (Ab). Nbs are derived from HcAb having one VHH, whereas mAb having two chains (heavy and light) gives rise to scFv.

For greater tissue penetration, both cAbs and HcAbs can be further broken down into smaller antigen-binding subunits. Some IgG modifications often used for these purposes are the VH and VL domains fusion via linker peptides to single-chain variable fragments, the production of independent autonomous human VH fragments, and Fc region cleaving to Fab fragments (25). However, because VHH domains derived from HcAbs are 1/10th of the molecular size of an IgG molecule (Nbs: 15 kDa and IgG: 150 kDa), they may be expressed as recombinant proteins called “Nbs.” Three of the antigen-binding loops that are commonly referred to as the VH and VL domains are fused via linker peptides to single-chain variable fragments as complementarity-determining regions, in addition to the four conserved framework regions that make up Nbs. Conversely, the extremely long CDR3 forms protrusions that aid in reaching cryptic epitopes frequently hidden from cAbs, granting the paratope its convex shape (24, 26).

### 3.2 Structural and functional characteristics

Through strategic substitutions that introduce more hydrophilic amino acids, VHHs gain improved solubility and prevent unwanted interactions between variable domains. This modification allows for a notably longer CDR3 region, which in turn creates a more diverse paratope than what is typically found in conventional antibodies. Although, in essence, the structure of their VH domain is similar to that of conventional antibodies, highly significant distinctions can be observed in the framework region 2 (FR2) and complementarity-determining regions (CDRs) of these molecules (27). One of the main factors for their wide antigen-binding repertoire, as well as their high solubility and resistance to aggregation, is the hydrophilicity of their FR2, which is an intrinsic property. In addition, these molecules can maintain their stability under the conditions of enzymatic digestion and pH changes, indicating considerable potential for oral delivery and other novel routes of administration in therapy (28).

Alternatively, a single immunoglobulin G (IgG) antibody of typical structure weighs approximately 150 kDa and has four polypeptide chains. The two heavy chains and two light chains are covalently attached to each other. Each heavy chain comprises one variable (VH) and three constant (CH1, CH2, CH3) domains, whereas each light chain consists of one variable (VL) and one constant (CL) domain. As Nbs are the structurally simplest forms, they have significant advantages over regular antibodies; they can retain their functionalities even in harsh environments, can go deeper inside tissues, are very soluble, and have the feature of recognizing specific, most probably hidden epitopes. They prefer to interact with non-planar surfaces like the grooves and cavities on antigens, thus allowing extremely accurate targeting of these sites (27). The set of properties that Nbs possess is very convincing. They can break the barriers of ordinary antibodies since they are easily bio-conjugated in order to elevate their effectiveness, adjust their pharmacokinetics, and facilitate more areas of use. Their physical robustness is beyond comparison; for instance, they stay stable for a long time at +4 °C and −20 °C, can tolerate high temperature (60 °C–80 °C) and extreme pressure (500–750 MPa), and, at the same time, are not affected by chemical denaturants like guanidinium chloride and urea. In addition, they show resistance to proteolytic enzymes and can coexist with a broad pH range (3.0–9.0), without a significant decrease in their antigen-binding capability. It is worth noting, however, that some aggregation of Nbs has been observed during heat denaturation processes (29). Some Nbs have demonstrated the capacity to cross the blood–brain barrier, making them promising candidates for the treatment of disorders affecting the central nervous system, especially those that have been specially engineered or chosen for this purpose (**Table 1**). However, CNS potential is limited by surface charge and rapid renal clearance. They can be fused with Fc domains or attached to polyethylene glycol as ways to enhance their pharmacokinetics (33). For instance, the kidneys quickly remove small Nbs without an Fc region that have a molecular weight of approximately 15 kDa after injection. Especially when radio-labeled Nbs are utilized for imaging or therapeutic purposes, this quick renal clearance may result in high kidney accumulation and an increased kidney radiation dose (34).

**Table 1:** Comparative characteristics of conventional antibodies and Nbs.

Feature	Conventional antibodies (IgGs)	Nanobodies (Nbs)	Functional implications	References
Thermal stability	Lose function at high temperatures below 90 °C	Retain structural integrity and function up to ~90 °C	High thermal tolerance allows easier storage, formulation, and delivery in diverse conditions	Lin et al. (30)
Molecular size	Large (~150 kDa), limiting access to compact epitopes	Small (~15 kDa), enabling recognition of hidden or recessed targets	Smaller size improves tissue access and binding to targets in vascular endothelium	Kang et al. (29)
Route of administration	Not compatible with nasal or aerosol routes	Suitable for inhaled or nasal formulations due to robustness	Enables non-invasive administration, potentially useful in pulmonary or vascular inflammation	Lodge et al. (31)
Immunogenic potential	Low but possible immune reactions, even for humanized antibodies	Generally, it is less immunogenic; certain motifs can still induce ADA formation	Lower immunogenicity supports safer long-term treatment but requires careful design	Lin et al. (30)
Tissue distribution	Poor penetration into dense tissues and barriers like the BBB	Capable of crossing the BBB and reaching interstitial spaces	Enhances targeting of vascular and neural inflammation sites	Manrique-Suárez et al. (32)
Solubility and stability	Stable and soluble; risk of aggregation at very high concentrations	Highly soluble, though some variants may aggregate under stress	Greater solubility facilitates high-concentration formulations for therapeutic delivery	Manrique-Suárez et al. (32)
Circulatory half-life	Naturally long, but variable with pharmacokinetic factors	Rapid clearance: half-life extended via Fc fusion or PEGylation	A short half-life allows rapid clearance but may require modification for sustained effects	Kang et al. (29)

### 3.3 Comparison of Nbs with other therapeutic fields

Although their benefits vary depending on the situation, Nbs have a number of unique characteristics that make them appealing for targeted cardiovascular applications as compared to traditional monoclonal antibodies and other therapeutic platforms. Because of their tiny molecular size, they can effectively penetrate tissues and reach sterically restricted or cryptic epitopes that full-length antibodies might find more difficult to access. Nbs typically offer excellent binding specificity while remaining flexible enough to be incorporated into multivalent, bispecific, or fusion constructs, in contrast to many small-molecule medicines that may exhibit weak target selectivity and off-target effects. Similarly, Nbs combine excellent physicochemical stability with relatively simple recombinant manufacturing, in contrast to siRNA and aptamer-based techniques, which frequently require specialized delivery systems or are vulnerable to stability-related problems. However, these advantages come with significant drawbacks (35).

Despite being useful for molecular imaging by lowering background signal, their low molecular weight causes quick renal clearance and a brief circulation half-life, which may limit their therapeutic usefulness. Several engineering techniques, such as Fc fusion, PEGylation, albumin-binding domains, and multivalent designs, have been developed to increase pharmacokinetic qualities while maintaining target specificity in order to overcome this constraint. Therefore, the biological target, the desired clinical application, and the balance between tissue accessibility, target engagement, and systemic exposure should be taken into consideration when choosing Nbs over alternative therapeutic or diagnostic techniques (36).

Nephrotoxicity may result from long-term renal retention of Nbs, particularly when they are radio-labeled. Such aggregation produces strong renal signals that make it challenging to see molecular targets in the surrounding regions. To lower renal absorption, various approaches have been devised. For instance, renal absorption of radio-labeled biomolecules can be decreased by co-injection with gelofusin or positively charged amino acids like lysine (37). Nbs have also been used with this method. Removing the affinity tags that are frequently employed for purification is another strategy. These tags, which are usually myc or (His)6 tags and are composed of negatively and positively charged amino acids, respectively, can have a direct effect on the degree of renal accumulation of the nanobody (38). Renal uptake may be reduced by PEG moiety attachment. The availability of PEGs in various sizes makes it easy to screen and adjust the desired construct to maximize its imaging properties. Using nanobody constructions with varying PEG moiety sizes, Rashidian et al. demonstrated a clear relationship between the size of the PEG moiety and the quantity of tracer building up in the kidney (39).

### 3.4 Generation and therapeutic design of Nbs

#### 3.4.1 Nbs generation process

Immune, naive, or synthetic libraries are used to create antigen-specific Nbs (40). Common methods for creating immune libraries include immunizing camelids with the target antigen at least four times over 2 months or, in cases where antigen availability is restricted, using transgenic mice that are designed to produce HCAbs (41). Following vaccination, lymphocytes are separated to extract mRNA and synthesize cDNA (42). The VHH sequence is amplified using a two-step PCR process, which uses primers that target restriction enzyme sites after the leader moves to the CH2 domain, resulting in smaller amplicons because the CH1 domain is absent. The amplicons are converted into an expression system, usually *Escherichia coli*, but they can also be used with yeast or mammalian systems after being ligated into vectors (43). At least 10 distinct transformants are present in an ideal immune library (40).

Phage display is the next method used to select specific Nbs. M13 phages infect Nb library cells to produce phages that show the Nbs fused to coat proteins. Triethylamine (pH 10) is used to elute positive binders after these are added to microtiter plates coated with the target antigen. Weak or non-binding clones are eliminated by repeated biopanning cycles (44). Strong binder phages are transfected into *E. coli*, ampicillin-cultured, and ELISA-validated before sequencing. High-affinity Nbs are then found using surface plasmon resonance (SPR), which measures the association (*k*_on_) and dissociation (*k*_off_) rates on an antigen-coated SPR chip. Dissociation constants in the nano/picomolar range and *k*_on_ = 10^5^–10^6^ and *k*_off_ = 10^−^²–10^−4^ are ideal for kinetics. Glycerol, glucose, and ampicillin are added to the culture media to create glycerol stocks of Nb-expressing *E. coli* WK6 for long-term storage (45). Future Nb production is made possible by these stocks (46).

#### 3.4.2 Therapeutic design of Nbs and respected applications

##### 3.4.2.1 Structure-based designs

Structure-based design of Nbs entails logical engineering and modification to improve stability, lower immunogenicity, increase binding affinity, adjust biophysical characteristics, or customize for particular uses. To stop tau aggregation associated with Alzheimer’s disease, for instance, sequences of fibril-capping amyloid inhibitors (WIW, VDW, W3) were grafted onto a previously described nanobody CDR-H3 scaffold (PDB ID: 6HEQ) (47). Furthermore, a bispecific nanobody was created that combines a blood–brain barrier targeting nanobody (IR5, a nanobody that targets type 1 insulin-like growth factor receptor) with the WIW tau capping nanobody inhibitor connected by a flexible linker (Gly4Ser)3. This nanobody showed enhanced blood–brain barrier penetration, indicating a potentially effective method of preventing prion-like tau seeding in neurodegenerative diseases (48).

Two essential components of synthetic nanobody library design are CDR design and framework selection. The VHH framework, which was generated from the very stable recombinant anti-chicken lysozyme nanobody cAbBCII10, was chosen. CDR-H1 and CDR-H2 preserved the constant length of cAbBCII10, whereas CDR-H3 had a 14-unit loop to create a convex binding site topology. Positions for randomization were found using the crystal structure study of cAbBCII10 as a guide, modifying codon use to guarantee stability (**Figure 2**). This involved removing destabilizing amino acids, highlighting polarity at solvent-exposed sites, and keeping particular amino acids in strategic locations to improve stability. Other stable frameworks with varying CDR-H1–3 lengths could employ this design approach (49).

**Figure 2:**
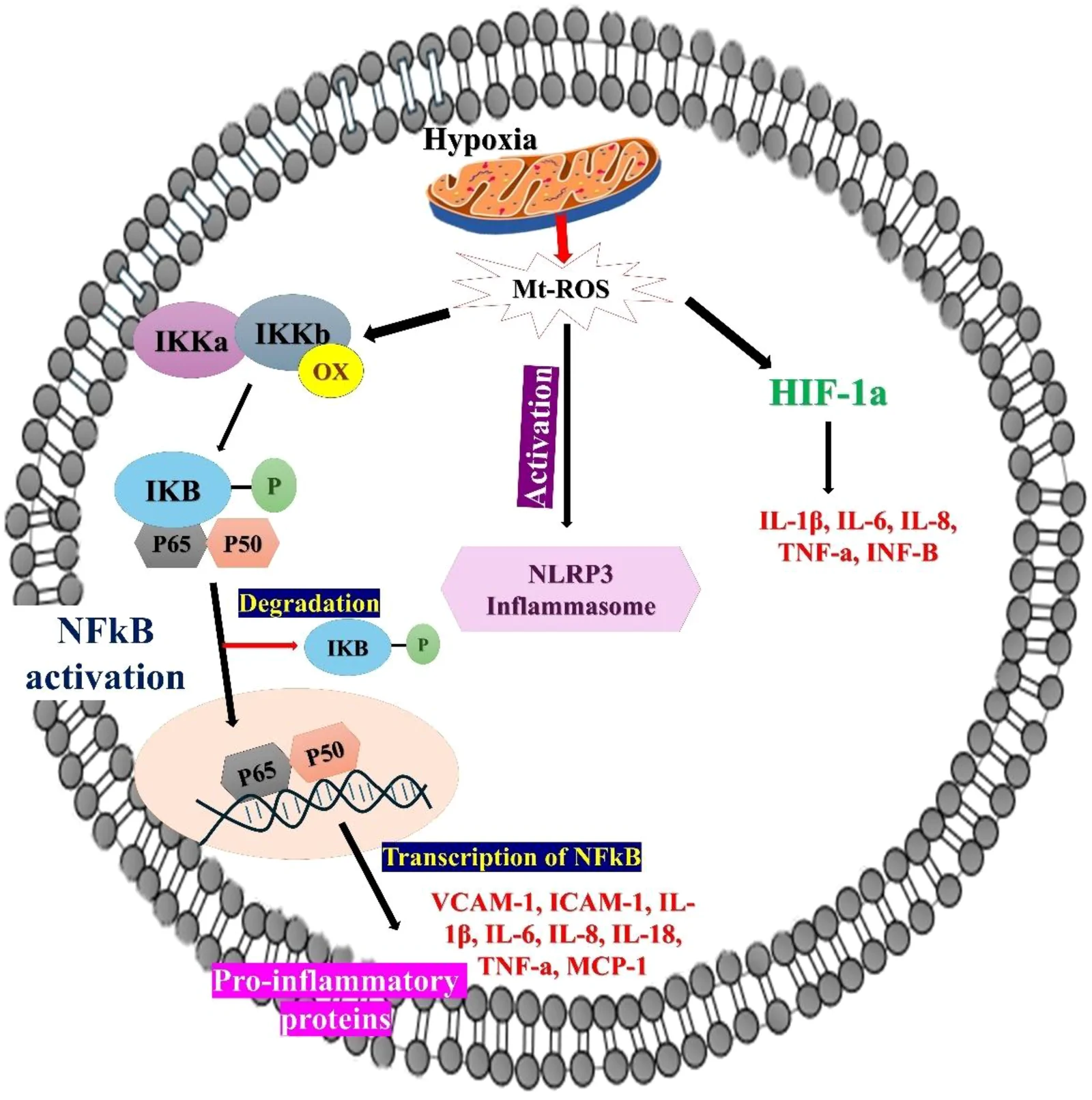
The role of ROS in activating pro-inflammatory signals is involved in causing endothelial dysfunction. Under hypoxia conditions, mitochondria generate O^−^ species that cause activation of three main reactions. First is NLPR3 inflammasome activation, second is HIF-1a activation that in turn activates cytokines, and the third one is NF-κB activation that activates pro-inflammatory proteins.

##### 3.4.2.2 Computational affinity maturation

To find mutations, changes, or non-natural amino acid incorporations that improve nanobody binding affinity while preserving specificity and stability, computational techniques allow the exploration of a large sequence and structural space (50). Based on the sequences of Nbs, a recently created deep-learning model called NanoBERT may be able to predict biologically plausible mutations in Nbs (51). Homology modeling was used to create the three-dimensional structure of a nanobody that targets CD20, a phosphoprotein that is abundantly expressed on B cells in non-Hodgkin lymphomas. Molecular docking simulations were then performed to examine the nanobody’s interaction with CD20 (52).

##### 3.4.2.3 Other approaches

It became common practice to humanize lead Nb for medicinal use to prevent potential immunological problems when administered to humans. The four VHH characteristic amino acids (mostly F/Y42, G/Q49, R50, and G52) found in framework region 2 should be preserved during this humanization attempt. Sadly, the remaining amino acids are dispersed across the gene and should be mutagenized to produce a sequence that resembles a human VH sequence (53).

When the lead Nb coding sequence is genetically fused with another gene, it produces precisely the intended construct when expressed. Since the paratope is likewise found at this end of the domain, it is preferable to keep the Nb at the 5′ end of the construct so that the N-terminal end of the Nb will be solvent-exposed. The fused genes are regularly separated by a linker, with the natural hinge of human IgA for its protease resistance or (Gly4Ser)3 for its improved flexibility between the fusion partners being preferred spacers (54). A bivalent construct created by fusing the coding sequences of two identical Nbs will probably exhibit an increased functional affinity because of avidity effects. A biparatopic Nb construct that forms a clamp on the Ag is created when two Nbs fuse and bind to non-overlapping epitopes on the same Ag. It may be essential to clone the Nbs in a different order and test various linker lengths for the best clamping. For bispecific T-cell engagers (BiTEs), where, for instance, an Nb against CD19 of B cells is fused with an anti-CD3 Nb targeting T cells, the fusion of two Nbs recognizing distinct Ags will bring the two cognate Ags closer together (55).

### 3.5 Imaging modalities and delivery format

#### 3.5.1 Imaging modalities

##### 3.5.1.1 SPECT

Single-photon imaging exploits the γ rays emitted by a radioactive atom. A y-emitting element with an energy of 100–250 keV is ideal. The spatial resolution of single-photon imaging is on the order of 8–10 mm, although somewhat better resolution can be obtained with recently developed special-purpose imaging systems (56). ^99m^Tc, ^123/131^I, ^111^In, and ^155^Tb are the four radionuclides suitable and commonly used for SPECT imaging; only two of them have been used with Nbs: ^99m^Tc and ^111^In (57). A few examples describe nanobody-based SPECT imaging using ^111^In as a radionuclide: Chatalic et al. developed an anti-PSMA nanobody conjugated to the metal chelator diethylenetriaminepentaacetic acid (DTPA), which showed good tumor targeting and minimal uptake by the non-targeted organs (58). Bala et al. made use of ^111^In to compare the pharmacokinetics of the imaging agent using the various tracers in conjunction with VCAM-1 targeting nanobody: ^68^Ga, ^18^F, ^111^In, and ^99m^Tc. The success of ^99m^Tc as a radionuclide for SPECT imaging can be attributed to both its easy radio-labeling chemistry and its advantageous nuclear decay properties. This has made it possible to label a variety of biomolecules (59). For instance, Nbs having a (His)6 tag can be directly labeled with ^99m^Tc(CO)3 without the need for further modifications. Moreover, the generation of ^99m^Tc is broadly accessible due to the extended half-life of ^99^Mo and the low cost of the ^99^Mo/^99m^Tc generator (60). The LOX-1 receptor was targeted in the atherosclerosis (ApoE-deficient mice) model using ^99m^Tc-labeled LOX-sdAb Nb (61).

##### 3.5.1.2 Positron emission tomography

Because positron-emitting radioisotopes are widely available and positron emission tomography (PET) has a higher spatial resolution and sensitivity than its single-photon cousin, it is seen to be a more appealing non-invasive imaging method (62). Nanobody-based PET tracers have been used as imaging agents to assess the presence or absence of cancer biomarkers in preclinical models. These biomarkers include HER2, HER3, CD20, EGFR, and HGF. These investigations’ findings demonstrated the nanobody-based probes’ significant tumor-specific absorption into the TME (63). The phase I clinical study using ^68^Ga-NOTA-labeled anti-HER2 nanobody in patients with breast cancer was a significant step toward the clinical translation of nanobody-based PET tracers (64).

##### 3.5.1.3 Infrared rays

SPECT and PET are more sensitive than infrared imaging. Compared to SPECT and PET, a significantly greater amount of the probe must collect in the targeted location for it to be seen. However, optical imaging has emerged as a feasible approach for *in vivo* imaging due to the recent invention of fluorescent dyes in the infrared and near-infrared area. In addition to the benefit of completely avoiding radioactivity, IR/NIR imaging is versatile, sensitive, quick, and affordable when compared to the radio-labeling strategy for SPECT and PET (65). Nowadays, *in vivo* imaging uses Nbs attached to these dyes (66). For instance, Bannas et al. employed Alexa Fluor 680 to image lymph nodes in ART2-TG and ART2^−/−^ mice utilizing a nanobody (s+16a) against the toxin-related ADP-ribosyltransferase ART2, an enzyme found on the surface of mouse T cells (67).

#### 3.5.2 Delivery systems

There is not much data available regarding how nanobodies were formalized and delivered to treat CVDs. So, how these nanobodies were formalized and delivered in other diseases has been mentioned as a reference that these nanobodies can be delivered using these formats for CVD treatment.

##### 3.5.2.1 As liposomes

A multivalent nanobody (Nb)–liposome platform that targets EGFR without the need for therapeutic medicines was created. Although steric hindrance caused a modest decrease in binding affinity, the anti-EGFR nanobody (EGa1) was successfully attached to PEGylated liposomes while retaining its antigen recognition capability. The approach showed increased tumor targeting by longer circulation and higher accumulation within tumor tissues, and it lowered cell surface EGFR expression by over 90% without the use of encapsulated medicines. However, because the liposomes contained no therapeutic ingredient, their overall anticancer activity remained restricted (68).

##### 3.5.2.2 As extracellular vesicles

In an extracellular vesicle (EV)-based delivery system, Kooijmans and colleagues used glycosylphosphatidylinositol (GPI) anchor peptides to display anti-EGFR Nbs on the EV surface. The targeting and binding efficacy of EVs toward EGFR-expressing cancer cells under both static and flow conditions was greatly enhanced by the successful surface expression of the Nbs. These results show that GPI-anchored nanobody-functionalized EVs have the potential to be a useful platform for targeted medication delivery in cancer treatment (69).

##### 3.5.2.3 As microbubbles

Nb-microbubble (μB) conjugates are a new platform for medication delivery and molecular imaging. Lipid microbubbles were successfully coated with biotinylated anti-eGFP and anti-VCAM-1 Nbs, exhibiting target-specific binding. The anti-VCAM-1-conjugated microbubbles demonstrated their promise as molecular ultrasonic contrast agents by successfully targeting VCAM-1 in both *in vitro* and *in vivo* settings. Additionally, this approach could improve targeted cancer treatment by enabling the regulated release of therapeutic medicines at tumor areas (70).

##### 3.5.2.4 As micelles

Through the increased permeability and retention (EPR) effect, micelles—nanosized carriers—improve the solubility, stability, and accumulation of hydrophobic medicines within tumor tissues. The capacity of micelles to target tumor cells and inflammatory regions is further enhanced by conjugating Nbs to their surface, which increases the uptake of encapsulated medications while decreasing off-target effects. This focused technique provides an effective method for improving medication delivery in inflammation and cancer treatment (71).

## 4 Vascular inflammation: nanobody-targeted molecules

Endothelial cells (ECs) are essential effector cells that trigger inflammation, control the body’s reaction to systemic inflammation, alter vascular function, and play an essential role in the etiology of vascular disorders (72). Dysregulated inflammatory responses can eventually lead to a variety of inflammatory disorders, such as obesity, hypertension, AS, autoimmune disorders, neurodegenerative conditions, diabetes mellitus (DM), sepsis, CVD, and cancer, even though inflammation serves as a self-defense mechanism (73). Therefore, a better comprehension of how ECs and inflammation interact is crucial.

### 4.1 Immune-mediated responses

#### 4.1.1 TLRs

Type I transmembrane proteins known as TLRs have been identified as important innate immunity mediators that trigger pro-inflammatory pathways in reaction to damage-associated molecular patterns (DAMPs) (74). TLRs stimulate the generation of pro-inflammatory modulators, such as cytokines, chemokines, and ROS, which cause vascular smooth muscle cell (VSMC) migration and proliferation by inducing the nuclear factor kappa B (NF-κB) signaling cascade. These are primarily seen in the vascular tissue and hypertensive microenvironment, where TLRs are constitutively expressed on several immune and nonimmune cells. At the same time, certain anti-TLR (toll-like receptor) treatments show a protective effect on microvascular integrity (75). The PD-L1/TLR7 Nb drug conjugate was designed in one study to improve the efficacy of anti-PD-L1 therapy. This conjugate induced an innate response, alleviating T-cell immunosuppression, and could reshape the tumor immune microenvironment (76).

#### 4.1.2 NLRP3

One of the primary initiators of the inflammatory response is the NLRP3 [nucleotide-binding oligomerization domain, leucine-rich repeat and pyrin domain-containing protein 3 (NLR family pyrin domain-containing 3)] inflammasome. The effector protein (caspase 1), adaptor [apoptosis-associated speck-like protein containing a caspase recruitment domain (ASC)], and sensor (NLRP3) make up this system. Pathogen-associated molecular patterns (PAMPs) and pro-inflammatory cytokines [like interleukin (IL)-1β and tumor necrosis factor-alpha (TNF-α)] mediate signal 1 (priming), while DAMPs, PAMPS, ATP, oxidized compounds, K^+^ efflux, Ca2^+^, and Na^+^ influx induce signal 2 (activation), which is necessary for NLRP3 activation. The inflammasome complex is activated, which is followed by the formation of caspase-1, and subsequently, IL-1β and IL-18 are produced as a result of these processes (77). The enzyme caspase-1 enables Gasdermin-D degradation after ROS-dependent oligomerization of this protein, which results in pyroptosis through membrane pore formation (**Figure 3**) (78).

**Figure 3:**
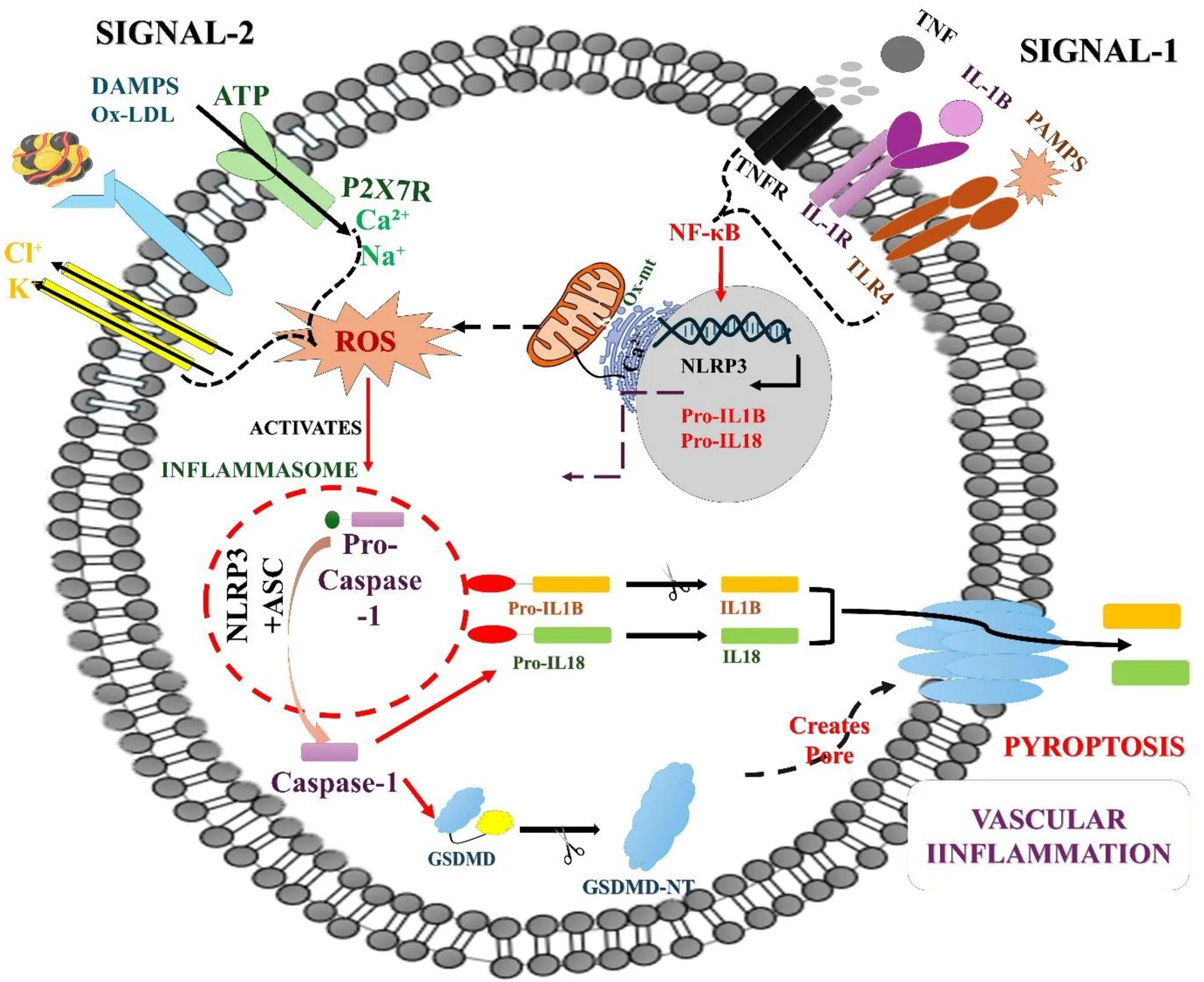
Activation of NLRP3 inflammasome in vascular inflammation. Priming (also known as “signal 1”) and stimulating (also known as “signal 2”) signals are necessary for the unique two-step process of NLRP3 activation. PAMPs (LPS) trigger the priming signal by binding to TLRs and causing the production of pro-IL-1β, pro-IL-18, and NLRP3. By binding NLPR3 and pro-caspase-1 to the adaptor protein ASC, a second activating signal is necessary for the assembly of the NLRP3 inflammasome. Potassium efflux via ion channels and cathepsin release via lysosomal degradation are examples of activating signals. After NLRP3 assembly and activation, pro-IL-1β and pro-IL-18 are further processed into their mature forms for secretion by caspase-1 through proteolytic cleavage. Furthermore, GSDMD is cleaved by caspase-1, leading to the creation of plasma membrane pores and pyroptotic cell death.

The NLRP3 inflammation response becomes active when excessive lipid accumulation occurs, which leads to plaque necrosis (79). The activation of NLRP3 in macrophages leads to atherosclerotic inflammation that develops into AS (80). NLRP3 inflammasome activation leads to IL-1β and IL-18 production, which promotes atherosclerotic plaque development and instability (81). Wan and his colleagues employed the NLRP3 silencing technique to reduce atherosclerosis and stabilize atherosclerotic plaques in diabetic atherosclerosis models by inhibiting NLRP3 inflammasomes and reducing the expression of ICAM-1 and VCAM-1 in the intima (82). NLRP3 inflammasome-related gene expression levels were higher in patients with symptomatic lesions, according to the findings (83). NLRP3 inflammasomes activate through multiple triggers, which include lysosomal breakdown, reactive oxygen species generation, mitochondrial dysfunction, and ion concentration changes (84). In *in vitro* and *in vivo* studies, Panx1-targeting Nbs exhibit anti-inflammatory properties and block Panx1 channel function by reducing IL-1B expression, which in turn was found to alter the expression of NLRP3 inflammasome (85).

### 4.2 Cytokine networks

#### 4.2.1 Interleukin-6

Production of IL-6 in CVD occurs via several cell types, including macrophages, monocytes, endothelial cells, VSMCs, and fibroblasts (86). The activation of TLR leads myeloid cells to produce IL-6 together with IL-1β and TNF-α. The activation leads to a rapid 6-fold increase in IL-6 production, which triggers an extensive positive feedback mechanism (87). IL-6 then attaches itself to the membrane-bound IL-6 receptor (IL-6R), found in a variety of cells, including leukocytes and endothelial cells. The Janus kinase/signal transducer and activator of transcription (Jak/Stat) pathway is one of the intracellular signaling pathways that are activated by the IL-6/IL-6R complex. Because it enables IL-6 to activate cells without IL-6R expressed on their membranes, soluble IL-6R is crucial for trans-signaling (86). Inhibition of T-cell apoptosis, recruitment of inflammatory cells, and inhibition of regulatory T-cell differentiation are among IL-6’s pro-inflammatory activities (88). There is a correlation between the levels of C-reactive protein (CRP) and IL-6, which is a crucial mediator for acute-phase reactions (89). A study was conducted where the cs130-TNF^VHH^Fc Nb conjugate was designed to target the IL-6/sIL-6R signaling pathway (90).

#### 4.2.2 Tumor necrosis factor-α

TNF-α is produced by both macrophages and cardiac myocytes, and it can prolong inflammation in both autocrine and paracrine ways (91). This cytokine is also produced by type 1 T-helper (Th1) cells. TNF-α production triggers a series of pro-inflammatory signaling events that can both protect and damage tissue. This cytokine has two primary receptor types, TNFR1 (p55) and TNFR2 (p75), which cause distinct signaling cascades due to differences in their intracellular domains. TNFR1 activation is associated with adverse effects, whereas TNFR2 activation plays a role in defense mechanisms (92). TNF-α’s main function in relation to cardiac tissue is to cause myocytes and endothelial cells to undergo apoptosis. It recruits neutrophils and macrophages, plays a part in calcium dysregulation, and increases the production of nitric oxide, all of which contribute to oxidative stress (93). The soluble receptor sTNF-RII is a significant endogenous modulator of TNF-α, and IL-10 inhibits TNF-α secretion (94). A study was conducted where the cs130-IL-12/23^VHH^Fc Nb conjugate was designed to target TNF-α (hTNF-α) or the IL-12/IL-23 signaling pathway (90).

#### 4.2.3 Interleukin-1 family

The pro-inflammatory cytokines contributing to heart disease development include IL-1α, IL-1β, and IL-18. IL-1β acts as the primary circulating form of IL-1 that rises during disease, but IL-1α is present generally in epithelial and mesenchymal cells, including myocardial cells, until cells undergo injury or death. As a result, IL-1β has become one of the most widely studied cytokines as a therapeutic target for various heart diseases (95). Monocytes, neutrophils, and macrophages are the significant producers of IL-1β and IL-18, which accumulate in the cytoplasm till they are activated by caspase-1, nucleotide-binding domain, and NLRP3. On the other hand, IL-37 and IL-38 lower the expression of these pro-inflammatory cytokines, even though they are also members of the IL-1 family (96). Endogenously generated IL-1 receptor antagonist (IL-1Ra), also known as anakinra, hinders the biological processes of IL-1α and IL-1β by attaching to the receptors and preventing their association (97).

#### 4.2.4 Interferon-γ

The cells that produce interferon-γ (IFN-γ) are classified into four groups: natural killer (NK) cells, macrophages, and CD4^+^ and CD8^+^ T lymphocytes. On top of that, IL-18 and IL-2 can also induce the production of IFN-γ (98). When IFN-γ is cleaved to its active form, it stimulates the build-up of low-density lipoprotein (LDL) in macrophages and initiates signaling via the Jak/Stat pathway. Instead, IFN-γ stimulates smooth muscle cells to express scavenger receptors and activates endothelial cells to increase the number of adhesion molecules, thus enabling the cells to migrate into the arterial intima and initiate the inflammatory process (99).

### 4.3 Chemokine and leukocyte recruitments

The cytokine class known as chemotaxis or chemokines functions as a chemoattractant to direct cellular movement are essential for developing embryos, regulating cell differentiation, survival, and proliferation. Chemokines have also emerged as critical players in developing atherosclerosis and vascular inflammation (100). The two major pathways through which chemokines activate cellular responses include seven-transmembrane G-protein-coupled receptors, also called “classical” chemokine receptors, and atypical chemokine receptors, ACKRs, which have defects in G-protein signaling. Investigators study the roles of chemokines and their receptors in atherosclerosis development by various approaches, including genetic and pharmacological methods (101).

#### 4.3.1 CCL2

The inflammatory chemokine CCL2 known as monocyte chemoattractant protein-1 (MCP-1) functions through its receptor CCR2 (101). CCL2 causes endothelial dysfunction through two mechanisms: it increases endothelin 1 production, and it activates E-selectin and ICAM-1 expression, which results in vasoconstriction, vascular remodeling, and chronic inflammation (102). People with elevated CCL2 blood levels have a higher risk of cardiovascular death (103). Clinical trials using colchicine to block the CCL2–CCR2 axis have proven to be an effective anti-inflammatory therapy for CVD prevention (104). The receptor–ligand complex of CCL2 binds to CCR2, which enables dimerization and internalization of monocyte chemoattractant protein-1-inducible protein-1. The receptor–ligand complex activation triggers multiple intracellular signaling pathways, including JAK/STAT, PI3K/MAPKs, and PI3K/Akt/ERK/NF-κB, which lead to CCL2, IL-1, and TNF gene expression (105). The study of Md Irfan and his colleagues demonstrated that Ang II-induced vascular remodeling and inflammation were decreased in CCR2 receptor-deficient mice, CCR2^−/−^ and BMT-CCR2^−/−^ with improved arterial wall macrophage infiltration (106).

#### 4.3.2 CCL5

The chemokine CCL5 (RANTES) stands as a major chemokine that T cells, endothelial cells, VSMCs, and macrophages release (107). Research using *in vivo* and *in vitro* methods demonstrates that blocking CCL5 or its primary receptor CCR5 leads to decreased atherosclerosis development and reduced vascular inflammation in lipodystrophy patients and perivascular adipose tissue inflammation in hypertension patients (108, 109). The CKLF1/CCR5 axis activates NF-κB/NLRP3 signaling after cerebral ischemia, which results in cell death (110). The blocking of CCR5 receptors during *in vivo* studies demonstrated their essential role in CVD development (111). The *in vitro* exposure of T cells and macrophages to aldosterone resulted in a small increase in CCL5 production, which remained higher than endothelial cell production. CCL5 exposure to mesenteric arteries and aortic tissue resulted in endothelial dysfunction, which suggests that CCL5 causes vascular damage through direct vascular effects or by modifying immune system function (112).

#### 4.3.3 CXCL8

One of the main causes of AS is the oxidation of LDL, which triggers the release of CXCL8, also called IL-8, and is interpreted by the immune system as a warning sign (113). According to one study, an siRNA for transmembrane protein 98 (TMEM98) suppresses the expression of intercellular adhesion molecule 1 (ICAM-1) on ECs, thereby preventing CXCL8-mediated leukocyte adhesion to the endothelium. Additionally, it suppresses the AKT/GSK3β/Cyclin D1 signaling pathway, which prevents vascular SMCs from proliferating and migrating. The CXCL8–miR-183–ABCA1 pathway functions as a transitional mechanism for foam cell development in AS, according to research that demonstrated CXCL8 triggers miR-183 expression, which leads to ABCA1 downregulation and reduced cholesterol export (114). The formation of foam cells through ABCA1 inhibition by CXCL8 leads to potential changes in AS development.

### 4.4 Endothelial adhesion molecules and the role of NF-κB

Because VCAM-1 contributes to the selective adhesiveness of lymphocytes and monocytes through the expression of counterreceptor very late antigen (VLA)-4, it has been considered an essential component of the endothelial activation cascade (**Table 2**) (119). By promoting leukocyte adhesion and migration, ICAM-1 is intimately involved in the interaction of endothelial cells and monocytes via its ligands, lymphocyte function-associated antigen-1, and macrophage-1 antigen (120). All P-, L-, and E-selectins are found on the surface of white blood cells, platelets, and endothelial cells, respectively. Endothelial cells’ Weibel–Palade bodies’ membranes are where P-Selectin, the biggest member of the family, is located. The binding of its main ligand, P-selectin glycoprotein ligand-1, present on the surface of leukocytes, demonstrates the involvement of P-selectin in leukocyte adhesion and rolling on the endothelium; however, there is also compelling evidence that it operates additionally as a signaling entity within the endothelium. E-selectin is essential for leukocyte trafficking and inflammatory responses because it can delay leukocyte rolling, which can result in leukocyte arrest. NF-κB attachment to the regulatory domains in its promoter is necessary for its upregulation (121). Both molecules are present on endothelial cells of atherosclerotic plaques. Mice with a deficiency in P-selectin or E-selectin had attenuated AS (122). Finally, MCP-1 is the first known inflammatory chemokine that is secreted by monocytes and endothelial cells. Its role is to mobilize leukocytes in the direction of the sub-endothelium by binding to the CCR2 receptor, which in turn contributes to AS (123). According to animal research, MCP-1 or CCR2 overexpression causes the opposite effects, such as macrophage accumulation and an acceleration of atherosclerosis, whereas deficiency results in decreased lipid deposition and a slowing of the AS process (124).

**Table 2:** Adhesion molecules associated with the interaction between vascular inflammation and ED and their clinical implications.

Adhesion molecule	Ligand	Role	Clinical significance	Functional implications	References
P-selectin	PSGL-1	Promotes platelet–leukocyte aggregation and leukocyte–endothelial adhesion	High plasma P-selectin concentrations are predictive of early cardiovascular complications in patients with suspected coronary artery disease (CAD)	Plays a dual role in thrombosis and vascular inflammation, contributing to endothelial injury and plaque instability	Kaur et al. (115), Zhang (116)
VCAM-1	VLA-4	Facilitates firm adhesion of leukocytes to activated endothelium	Elevated baseline VCAM-1 levels are observed in healthy middle-aged individuals who later develop cardiovascular disorders	Key mediator in early atherogenesis through recruitment of monocytes to vascular endothelium	Kunutsor et al. (117)
MCP-1	CCR2	Directs monocyte migration toward inflamed endothelial tissues	Increased MCP-1 concentrations are associated with a higher incidence of both peripheral artery disease (PAD) and CAD, independent of traditional risk markers	Enhances macrophage infiltration and foam cell formation, accelerating plaque development	Blanco-Colio et al. (118)
ICAM-1	LFA-1, Mac-1	Supports tight adhesion and transmigration of leukocytes across the endothelial barrier	ICAM-1 expression shows a strong correlation with coronary and carotid atherosclerosis, regardless of other cardiovascular risk determinants	Promotes inflammatory cell infiltration and progression of vascular lesions	Kunutsor et al. (117)
E-selectin	ESL, PSGL-1	Initiates the rolling phase of leukocytes on activated endothelial surfaces	Elevated circulating E-selectin levels are linked to increased CAD and carotid atherosclerosis risk, even in the absence of classic risk factors	Serves as an early biomarker of endothelial activation and inflammation	Kunutsor et al. (117)

## 5 Endothelial dysfunction pathophysiology

A major factor in the development and course of many diseases is ED. It is characterized by a dysregulated release of endothelium-derived factors, which results in abnormal modulation of vascular growth, increased pro-inflammatory and pro-thrombotic states, and decreased vasodilatory capacity. By increasing vascular stiffness, decreasing arterial distensibility, and aggravating damage to the myocardium and vasculature, ED plays a vital role in the pathophysiology of AS in CVD (125).

### 5.1 eNOS uncoupling and ED

Often, the first mediator of ED is decreased NO synthesis and availability, which results from compromised eNOS expression and activity. Increased oxidative stress causes NO uncoupling, a process in which the production of superoxide rather than NO is driven by insufficient cofactor concentrations. This has detrimental downstream effects, including the production of a pro-oxidant peroxy-nitrite, which initiates mitochondrial dysfunction and ED (126).

Major vicious cycles of NO consumption can be the direct interaction of reactive oxygen species (ROS) with NO to form peroxynitrite (ONOO^−^), which then causes eNOS to be uncoupled, increases O_2_^•−^ and decreases NO production. In addition, ROS can inactivate eNOS by S-glutathionylation and also decrease eNOS gene expression; thus, NO production is further reduced (127). Moreover, ROS increases protein arginine N-methyltransferase (PRMT) expression, which converts L-arginine to ADMA and thus increases ADMA levels, and lowers dimethylarginine dimethylaminohydrolase (DDAH) activity, which leads to reduced asymmetric dimethylarginine (ADMA) degradation (128). In fact, reduction of NO levels and eNOS uncoupling can also result from ADMA, an endogenous NOS inhibitor. In the end, ROS converts BH4 to dihydrobiopterin (BH3•), thus reducing BH4 levels and leading to eNOS uncoupling, which in turn results in a decrease in NO bioavailability (**Figure 4**) (129). Depleted BH4 stores may also contribute to ADMA-induced oxidative stress, which would further cause eNOS uncoupling and increased superoxide production. Finally, because of leukocyte adhesion and the generation of inflammatory mediators, ADMA has also been shown to have a pro-inflammatory function (130).

**Figure 4:**
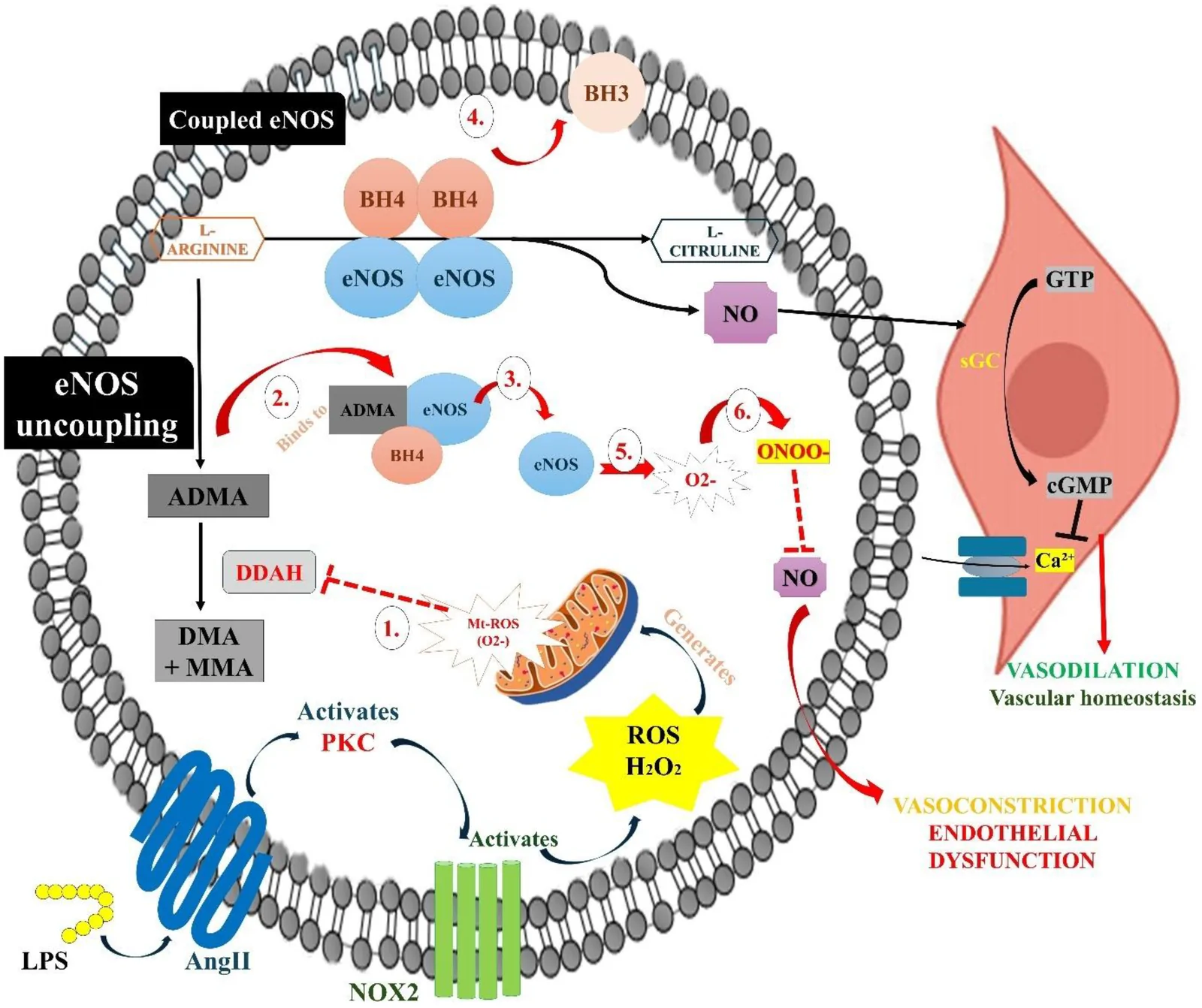
Role of oxidative stress on the generation of eNOS uncoupling leading to endothelial dysfunction. With the essential cofactor tetrahydrobiopterin (BH4), the eNOS uses oxygen and L-arginine (L-Arg) as substrates to produce NO and L-citrulline. When eNOS uncoupling occurs, NO is replaced by O_2_^·−^. Superoxide anion quickly oxidizes BH4 to BH2 during oxidative stress, which competitively replaces BH4 for heme binding and encourages eNOS uncoupling. The dihydrofolate reductase (DHFR) can then recycle BH2 into BH4. The L-Arg/ADMA ratio may drop as a result of oxidative stress, hence decreasing substrate consumption.

### 5.2 ROS and ED

Oxidative stress, by involving the NLRP3 inflammasome that contains pyrin domain (PYD), NF-κB, hypoxia-inducible factor-1 alpha (HIF-1a), nucleotide-binding domain (NBD), and leucine-rich repeat (LRR), stimulates inflammatory responses, thus leading to ED and AS progression at a faster rate. The inhibitory IKKB protein is in control of the cytoplasmic expression of NF-κB, a transcription factor that is in charge of the production of inflammatory cytokines. ROS can oxidize the IKK complex that phosphorylates IKB, thus leading to its degradation. As a result, NF-κB is activated and moves to the nucleus. By elevating the production of inflammatory mediators (such as IL-18, IL-8, IL-6, IL-1β, and TNF-α), adhesion molecules (ICAM-1 and VCAM-1), and chemokines (MCP-1), the activation of this pathway generates a pro-inflammatory microenvironment that causes endothelial injury and intimal thickening (131). Second, pro-inflammatory genes linked to HIF-1α are expressed when ROS stabilizes HIF-1α (132). Additionally, pro-IL-1β and pro-IL-18 are cleaved into their mature forms by caspase-1 of the ROS-stimulated NLRP3 inflammasome complex, which is made up of NLRP3, caspase-1, and the adaptor protein ASC. Leukocyte and monocyte migration in the initial phase of AS is mediated by the release of IL-1β, which stimulates the synthesis of adhesion molecules and chemokines (**Figure 2**) (133). For example, TNF-α causes endothelial cells to produce more inducible NOS (iNOS), activate NADPH oxidase, and produce mitochondrial ROS (mtROS) (134). As a result, ED, inflammation, and oxidative stress are all closely related processes.

### 5.3 ox-LDL-induced ED

One of the main causes of endothelial cell activation, dysfunction, and damage is oxidized LDL (ox-LDL). When the endothelium malfunctions, the vascular endothelium’s vasodilatory capabilities are compromised, which results in disruption of the barrier, elevated vessel permeability, and elevated expression of leukocyte adhesion molecules (125). In the subendothelial layer, adherent monocytes will develop into macrophages. These macrophages will then absorb ox-LDL through scavenger receptors [such as CD36, lectin-like ox-LDL receptor-1 (LOX-1), and scavenger receptor A1] and become lipid-laden foam cells. Macrophage polarization, inflammation, and inflammasome activation can all be brought on by ox-LDL and its lipotoxic constituents, including lyso-phosphatidylcholine (LPC). Additionally, ox-LDL can stimulate platelet activation and aggregation (135).

#### 5.3.1 ox-LDL triggers endothelial barrier disruption and hyperpermeability

By preventing direct contact between circulating cells and the endothelium, the endothelial glycocalyx—which is made up of glycoproteins and proteoglycans—maintains the integrity of the vascular barrier. Glycocalyx degradation brought on by atherogenic levels of ox-LDL exposes the endothelial surface and encourages hyperpermeability and platelet adhesion (136). KLF2, a crucial transcription factor that controls tight junction proteins like occludin, is suppressed by ox-LDL at the molecular level (137). Its downregulation impairs barrier function by weakening intercellular junctions. Together, ox-LDL impairs the endothelial barrier’s molecular (KLF2 suppression) and structural (glycocalyx degradation) elements, resulting in ED and increased permeability—two crucial early stages of vascular inflammation and AS (138).

### 5.4 Angiotensin-II mediated mitochondrial damage and ED

The ECs’ Ang II type 1 (AT1) receptor is bound by the endocrine peptide hormone Ang II (139). A signaling pathway is started in the cytosol following AT1 receptor binding and activation, which results in decreased transcription of sirtuin-3 mitochondrial NAD-dependent deacetylase (SIRT3) in the nucleus and subsequently decreased SIRT3 protein in the cytosol. Consequently, SIRT3’s localization in the mitochondria is diminished (**Figure 5**). Superoxide dismutase 2, a mitochondrial antioxidant enzyme, is increased in acetylation (hyperacetylated) as a result of its decreased localization in the mitochondria, which inactivates it. In mice, this results in ED due to mitochondrial oxidative stress, which damages the mitochondria (140, 141).

**Figure 5:**
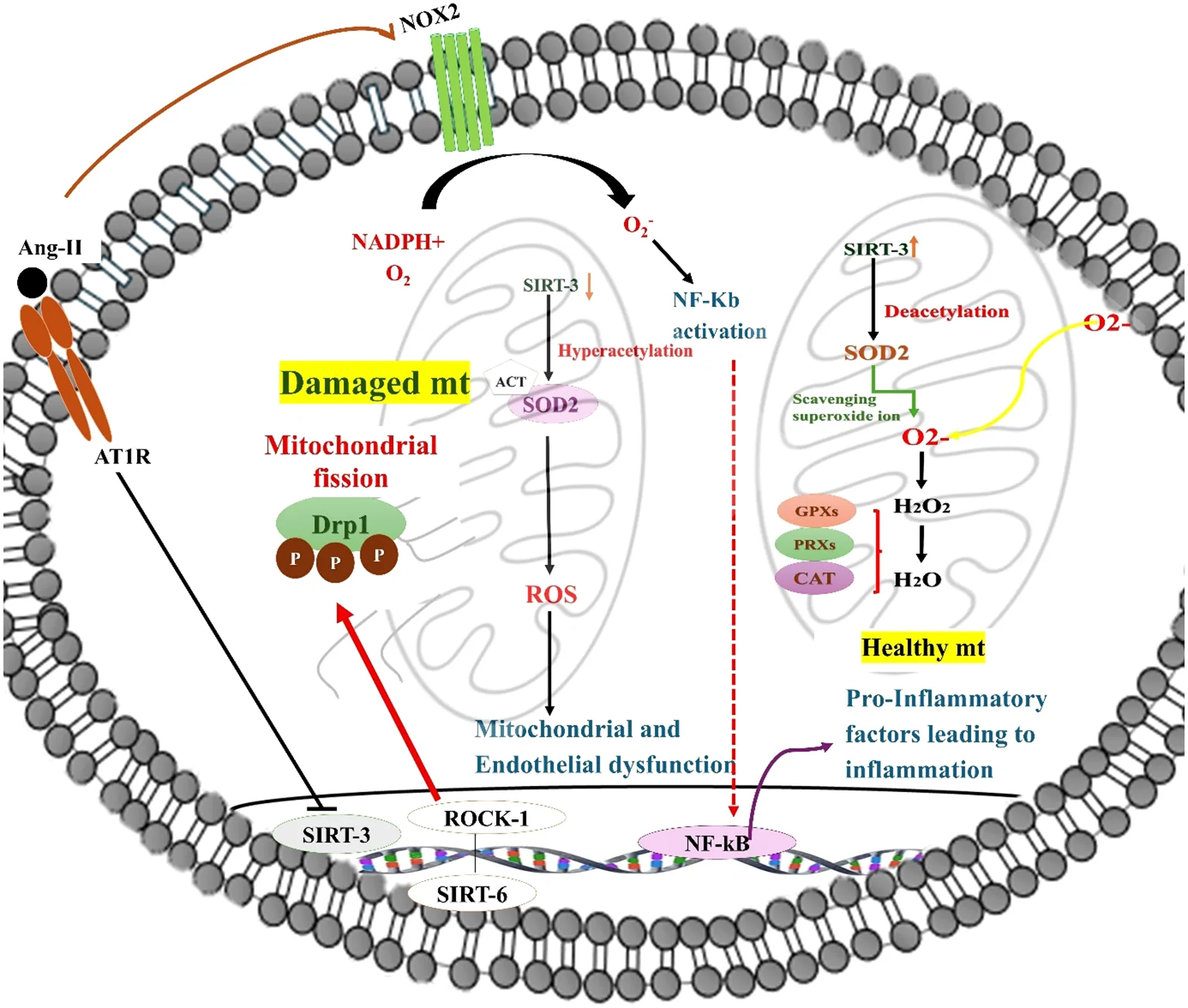
The role of Ang II-induced mitochondrial stress in inflammation and endothelial dysfunction. Reactive oxygen species (ROS) generation is increased when angiotensin II (Ang II) attaches to the AT_4_ receptor and activates NADPH oxidase (NOX). This reduces ATP, destroys mitochondrial DNA, and causes the release of mitochondrial ROS. Endothelial dysfunction and inflammation are caused by this vicious cycle of oxidative stress, which also activates pro-inflammatory transcription factors like NF-κB.

Alternatively, Ang II can activate nicotinamide-adenine dinucleotide phosphate (NADPH) oxidase 2 (NOX2), an oxidase enzyme on the plasma membrane of aortic ECs that generates O^2−^ *in vitro* and *in vivo* using an oxygen molecule (O_2_) and NADPH cofactor (142). The mitochondrial respiratory chain is then inhibited, and apoptosis/necrosis is induced as a result of O^2−^ interacting with nitric oxide (NO) to produce peroxynitrite (NO^3−^) in the cytosol (143). In rat aortic ECs, Ang II causes mitochondrial fission, which leads to endothelial dysfunction. Experimentally, it is suggested that Ang II increases Rho-associated protein kinase 1 (ROCK1/ROCK1) transcription and protein level by downregulating sirtuin 6 (SIRT6/SIRT6) transcription and protein level, which lowers histone deacetylation. Dynamin-related protein 1 (Drp1) becomes more phosphorylated and activated as a result of an increase in ROCK1 protein levels in the cytosol. Following its translocation to the mitochondria, Drp1 causes mitochondrial fission or dysfunction. Thus, mitochondrial fission in aortic ECs is linked to Ang II-mediated activation of the SIRT6–ROCK1–Drp1 axis, ultimately leading to endothelial dysfunction (144).

### 5.5 Platelet aggregation, von Willebrand factor, and ED

The endothelium can produce thrombin synthesis and activity inhibitors in healthy conditions, which helps to maintain the equilibrium between fibrinolysis and coagulation. However, ED and EC activation that follow vascular injury and inflammation cause coagulation factors to be upregulated, which results in a pro-thrombotic phenotype. The external route of coagulation can be initiated by activated EC expressing active tissue factor on the cell surface. Experimental data indicate that this process involves inflammatory mediators such as TNF-α, CD40 ligand, and other inflammatory cytokines (145).

Platelet mobilization is also a result of endothelial cell activation. The vascular endothelium, among other things, releases prostaglandins and NO to inhibit platelet adhesion and aggregation under physiological conditions. However, when endothelial activation and the ensuing vascular injury take place, the Weibel–Palade bodies of ECs release several mediators, including von Willebrand factor (vWF), angiopoietin-2, and P-selectin. Recently, it was divulged that platelet-derived vWF only marginally contributed to thrombus formation, while endothelial-derived vWF, an essential molecule found to be associated with platelet coagulation and activation, was in charge (146). Similar results were noted regarding the vWF-dependent atherosclerotic process (147). Inflammatory stimuli such as superoxide anions and cytokines cause vWF, an acute-phase reactant, to increase. Because of this, it contributes to both thrombosis and inflammation by encouraging aggregation and adhesion of platelets and the inhibition of proteolytic degradation of factor VIII, along with leukocyte mobilization, NETosis, and complement activation (148). **Table 3** shows the summary comparing candidate nanobody targets, disease context, intended application, evidence level, advantages, limitations, and translational status.

**Table 3:** Summary of nanobody targets in endothelial dysfunction and vascular inflammation, their application, pros and cons, and clinical and translational evidence.

NB target	Disease	Intended application	Clinical evidence	Benefits	Limitations (as NB targets)	Translational status	References
VCAM-1	ED and AS	Targeted delivery to inflamed endothelium; molecular imaging	Preclinical	Excellent target-to-background imaging contrast, quick tissue penetration, and high specificity for activated endothelium	Limited therapeutic validity and a short plasma half-life	Experimental	Kong et al. (149)
Pannexin-1 (PANX1)	ED and VI	Regulates vascular tone and modulates inflammatory pathways	Preclinical	A new endothelial target that may control vascular inflammation	Insufficient *in vivo* cardiovascular and mechanistic evidence	Early-stage research	Van Campenhout et al. (85)
von Willebrand factor (vWF)	Thrombotic thrombocytopenic purpura (TTP), thrombosis, and vascular disorders	Diagnostic detection of active vWF; antithrombotic therapy	Clinical (caplacizumab approved for acquired TTP)	High affinity, rapid target binding, first clinically approved nanobody	Clinical use currently limited to TTP rather than atherosclerotic CVD	Clinically approved (non-atherosclerotic indication)	Watanabe et al. (147)
TNF-α	VI	Anti-inflammatory therapy	Experimental (non-CVD studies)	High target specificity	No direct evidence related to CVD	Theoretical	Evavold et al. (78)
IL-1β	AS and VI	Cytokine neutralization	No	Potential biomarker as an inflammatory target	No direct evidence related to CVD	No	Griendling et al. (77)
IL-6/IL-6R	ED and VI	Cytokine inhibition	Experimental	Potential for cytokine inhibition and increased tissue penetration	No direct evidence related to CVD	Early-stage research	Aparicio-Siegmund et al. (88)
Oxidized LDL (ox-LDL)	AS	Initiates inflammatory signaling pathway, disrupts endothelial barrier function, and regulates high permeability of the vascular endothelial monolayer cells	No	Potential biomarker for plaque formation	No direct evidence related to CVD	No	W. Li et al. (137)
AT1R (angiotensin II type-1 receptor)	Hypertension and vascular remodeling	Receptor inhibition	No	Potential target in CVD	No direct evidence related to CVD	No	McMahon et al. (150)
ROS/NADPH oxidases	Oxidative stress and ED	Compromise the endothelium’s NO, worsening the intracellular environment, and raising intravascular permeability	No	Key regulators of endothelial injury	No direct cardiovascular approach was revealed	No	Yuk et al. (134)
CCL2/MCP-1	Monocyte recruitment in AS	Chemokine inhibition	Experimental (non-CVD studies)	Possibility of lowering the recruitment of inflammatory cells	Studies on cardiovascular nanobodies are still rare	Early-stage research	Banecki et al. (102)
CCL5/RANTES	AS and VI	Chemokine blockade	Experimental (non-CVD studies)	Can reduce plaque inflammation and leukocyte recruitment	Insufficient evidence in CVD study	Early-stage research	Döring et al. (112)

## 6 How do vascular inflammatory and endothelial dysfunctional factors cause atherosclerosis?

Endothelial cell damage and dysfunction are the first signs of AS (151). AS is encouraged by this damage because it increases vascular penetration and allows positively charged lipoproteins to flow through and accumulate in the negatively charged, proteoglycan-rich subendothelial cellular region. They experience oxidative modification there (152). During this stage, monocytes are guided to traverse the endothelium by upregulated expression levels of VCAM-1, ICAM-1, P-selectin, E-selectin, and MCP-1 (149). These monocytes change into macrophages inside the vessel wall by the localized action of macrophage-colony stimulating factor (M-CSF), exhibiting an anti-inflammatory or pro-inflammatory phenotype, depending on stimuli from the exterior microenvironment (153, 154). Scavenger receptors (SRs) like SR-A, CD36, and CD68 allow pro-inflammatory macrophages to absorb oxidized low-density lipoproteins (ox-LDL), which causes them to change into foam cells that are rich in cholesterol esters (155). Their initial immune function and capacity to eliminate dying cells may be compromised by the appearance of foam cells (156). Additionally, they can release IFN-γ, TNF-a, IL-1, MCP-1, platelet-derived growth factor (PDGF), IL-6, CCR2, CCR5, and other factors, which enhance intravascular inflammatory responses by attracting more immune cells (**Figure 6**). The apoptosis or necrosis of foam cells increases the risk of rupture at lesion sites by enabling the formation of a lipid core composed of cellular debris, cholesterol esters, and crystals (157, 158). VSMCs then move from the media to the intima and enter a proliferative state, generating ECM elements such as proteoglycans, collagen, and elastin (79). Luminal narrowing is the result of these processes, which also help to thicken the vessel wall and form a fibrous cap, giving the plaque stability (159).

**Figure 6:**
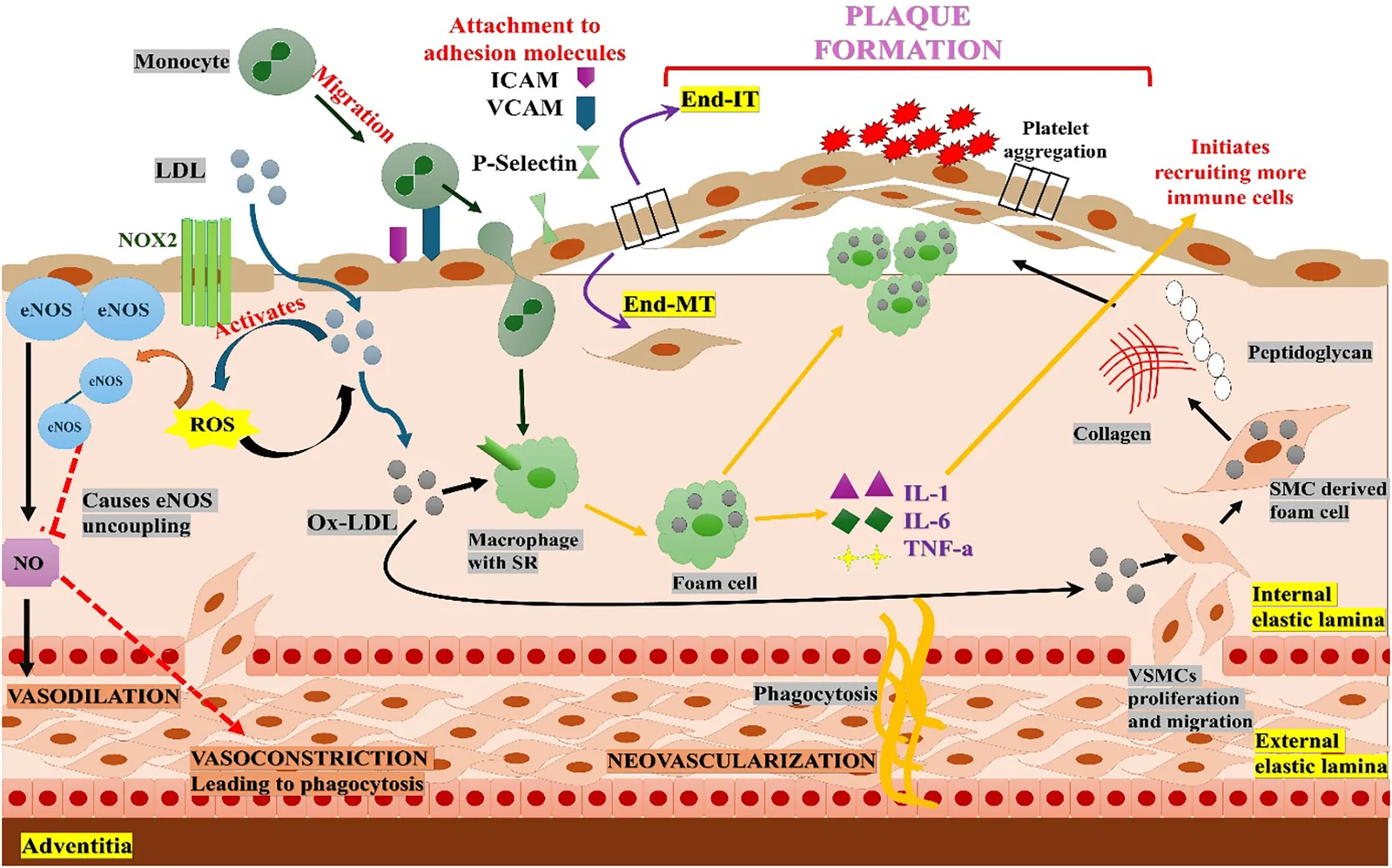
Role of vascular inflammatory and endothelial dysfunctional factors in causing atherosclerosis. LDL, when entered into the cell surface, activates ROS, which triggers two reactions. First is eNOS uncoupling leading to vasoconstriction, ultimately phagocytosis, and second is oxidation of LDL (Ox-LDL), which, when engulfed by macrophages, produces foam cells. These foam cells cause plaque formation and the secretion of pro-inflammatory factors that recruit more immune cells, initiating a cascade of inflammatory reactions.

## 7 Application of Nbs in treating vascular inflammation and endothelial dysfunction

Though several advancements have been made using nanobody technology, these advancements are in the field of oncology. Data regarding the role of Nbs in treating ED and vascular inflammation are very scarce. Therefore, we have mentioned various possible approaches in this section to use Nbs in dealing with vascular inflammation and ED (**Table 4**).

**Table 4:** Summary of molecular factors in endothelial dysfunction and vascular inflammation—nanobody evidence.

Factor	Role in vascular inflammation/endothelial dysfunction	Clinical evidence (yes/no)	Nanobody type and purpose (diagnostic/therapeutic)	Disease model	References
VCAM-1	Mediates leukocyte adhesion; endothelial activation marker; key in atherosclerotic lesion formation	Yes	Diagnostic—^99m^Tc-cAbVCAM1-5 (SPECT), ^18^F-FB-anti-VCAM-1 (PET). Enables molecular imaging of inflamed endothelium	ApoE^−^/^−^ mouse model of atherosclerosis, *ex vivo* imaging of human plaques	Broisat et al. (160)
Pannexin-1 (PANX1)	ATP release channel promotes leukocyte recruitment and inflammation in ischemia–reperfusion injury	Yes	*Therapeutic*—PANX1-blocking nanobody reduces infarct size and inflammatory infiltration	*Mouse myocardial ischemia–reperfusion* model	Van Campenhout et al. (85)
von Willebrand factor (vWF)	Mediates platelet adhesion and thrombus formation; elevated in endothelial dysfunction and microvascular injury	Yes	*Therapeutic (approved drug)*—*caplacizumab* (anti-vWF Nanobody^®^) blocks vWF–platelet interaction; used for aTTP	*Clinical*—acquired TTP patients; no CVD model use reported	Duggan (161)Jovčevska et al. (162)
TNF-α	Induces adhesion molecules, oxidative stress, and endothelial apoptosis.	Yes (preclinical)	*Therapeutic*—anti-TNF Nbs tested for anti-inflammatory and antitumor effects; not yet in CVD.	*Tumor models* (murine xenografts); *inflammatory disease* models. No CVD model has been published	Yoshiya Tanaka et al. (163)
IL-1β	Central mediator of vascular inflammation; triggers leukocyte infiltration, endothelial activation, and plaque instability	Yes	*Therapeutic (preclinical)*—IL-1β nanobody reduces inflammation and apoptosis under hypoxia	*Rat cardiomyocyte hypoxia model; in vitro cardiac injury* studies	Yoshiya Tanaka et al. (163)
IL-6/IL-6R	Drives acute-phase response; linked to vascular inflammation, thrombosis, and endothelial dysfunction	Yes	*Therapeutic (preclinical)*—IL-6R nanobody (ALX-0061) or bispecific nanobody fusion inhibits IL-6 trans-signaling	*Autoimmune/inflammatory disease* models (rheumatoid arthritis, systemic inflammation). No CVD model has been published	Zhou et al. (164)
Oxidized LDL (ox-LDL)	Activates endothelial cells, induces ROS and foam cell formation	No	No nanobody data available	–	–
AT1R (angiotensin II type-1 receptor)	Promotes vasoconstriction, oxidative stress, and endothelial inflammation via Ang II signaling	Yes (non-CVD)	*Diagnostic/structural*—Nbs are used to stabilize AT1R for crystallographic studies and molecular imaging (not therapy)	*Structural/imaging* studies; *no CVD or in vivo* therapeutic model reported	Wingler et al. (165)
ROS/NADPH oxidases	Drive oxidative stress, NO depletion, endothelial barrier dysfunction	No	No nanobody research has been published for ROS/NADPH oxidase inhibition in CVD or other models	–	–
CCL2/MCP-1	Monocyte chemoattractant: major driver of vascular inflammation and plaque growth	No	No published nanobody-based studies (diagnostic or therapeutic)	–	–
CCL5/RANTES	Recruits monocytes, T cells, platelets; promotes atherosclerotic lesion growth	Yes (non-CVD)	*Therapeutic (viral immunology)*—Nbs developed against the CCR5/CCL5 axis in *HIV/viral infection models*; none in CVD	*Viral infection/immunology* models	Bergkamp (166)

### 7.1 Nbs in diagnosing and treating vascular inflammation

#### 7.1.1 Anti-cytokines and anti-chemokines Nbs

Nbs have emerged as tools during the last 10 years to study the initial inflammatory processes of atherosclerosis through both diagnostic and therapeutic applications. AS is initiated with the release of inflammatory cytokines and chemokines, but data regarding Nbs’ role in inhibiting cytokines and chemokines are limited in AS models. So, Nbs’ role in targeting them in other diseases has been discussed to get an idea of how Nbs will target them in CVD models *in vitro* and *in vivo*. The anti-TNF-α antibody ozonorizumab is a 38-kDa trivalent Nb molecule. It is composed of one humanized anti-human serum albumin VHH Ab and two humanized anti-human TNF-α VHH Abs. Rheumatoid arthritis (RA) is largely caused by TNF-α, which also contributes to the persistent inflammation and joint deterioration that RA patients endure (163). By neutralizing TNF-α, ozoralizumab can reduce inflammation in RA patients, improving joint function and reducing symptoms. Both with and without MTX, ozoralizumab has a favorable safety profile and shows significant therapeutic effects in patients with active RA (167, 168).

As a ground-breaking myocardial repair method, scientists employed a ROS-degradable smart hydrogel to deliver IL-1β-VHH nanobody (IL-1β-VHH Nbs) locally. After a heart attack, this therapeutic gel was injected into the pericardium of animal models, where it easily spread over the surface of the heart, forming a stable epicardial patch right at the injured site. Nbs that are released from this hydrogel showed an impressive capacity to go through cardiac muscle tissue. Results show that the gel sticks well to the damaged parts of the heart; thus, it locally elevates the concentration of anti-IL-1β therapy. By blocking the IL-1β cytokine and leading to M2-type macrophage polarization in the infarcted areas, the anti-IL-1β hydrogel helps cardiomyocytes to be spared from apoptosis and also supports physiological cardiac regeneration. This study highlights the strength of such a comprehensive, synergistic approach to myocardial infarction treatment and unveils its significant potential to become a valuable helper in the healing of the heart that has suffered damage (169).

Anti-FGF-2 Nbs were one of the components of a separate experiment on psoriasis, a mouse model in which the condition was induced by imiquimod. Their use resulted in the reduction of the severity of psoriatic skin lesions, and it very effectively blocked the overmigration and proliferation of keratinocytes that were exacerbated by the disease, thus placing the treatment as one of the most probable first therapeutic candidates in the future. The Nbs normalized the inflammatory milieu by inhibiting the selected pro-inflammatory cytokines (IL-1β, IL-6, IL-23, and TNF-α), chemokines (CXCL1 and CCL20), and neutrophil infiltration as well. This strategy constitutes a new way of therapy by even more accurately targeting the FGF-2-dependent growth signaling pathway in psoriasis (164). Additionally, the study on chemokine-binding Nbs unveiled the definite sequence motif for the antigen-binding regions in their Nbs; hence, the researchers suggested that tyrosine sulfation might be the principal mechanism of their function. They hypothesized that if the Nbs were sulfated, the chemokine binding would be more efficient, and the selectivity would be higher because the post-translational tyrosine sulfation of receptors for chemokine affinity has been the most significant factor. Four Nbs (16C1, 9F1, 11B1, and 11F2) with tyrosine sulfation were produced by amber codon suppression. The sulfated 16C1 form showed substantially stronger binding to CCL2 chemokines than the non-sulfated one. This could be applied to inflammatory vascular diseases to target CCL2 chemokines (170).

#### 7.1.2 Nbs targeting adhesion molecules (anti-VCAM Nbs)

The research by Senders and colleagues showed that PET/MRI imaging can detect inflamed endothelium and early atherosclerotic lesions through the use of radio-labeled Nbs that bind to VCAM-1. They tested three nanobody radiotracers that were directed against distinct biomarkers of the advancement of AS: macrophage mannose receptor (MMR), lectin-like oxidized low-density lipoprotein receptor (LOX)-1, and vascular cell adhesion molecule (VCAM)-1. Because of their quick renal clearance, the three nanobody radiotracers showed short circulation times and accumulated in atherosclerotic plaques. In an ensuing PET/MRI study of the progression of atherosclerosis, the MMR nanobody was chosen to be labeled with gallium-68 (68Ga), a short-lived radioisotope with high clinical relevance. Dynamic contrast-enhanced (DCE) MRI was used to study neovascularization, T2-weighted MRI was used to study plaque burden, and 68Ga-MMR-PET was used to study macrophage burden over time. Additionally, fluorine-18 (^18^F)-labeled fluorodeoxyglucose (^18^F-FDG) was used to assess inflammation. All of the previously mentioned metrics increased as the illness worsened, and there was a correlation between the imaging signatures and histopathological characteristics (171). Correspondingly, scientists probed whether molecular imaging of VCAM-1 might be a practicable, non-intrusive substitute for identifying inflammation. They labeled an anti-VCAM-1 nanobody (^99m^Tc-cAbVCAM1-5) with a radioisotope and then applied single-photon emission computed tomography (SPECT) to visualize its injection in the livers of animal models of non-alcoholic fatty liver disease (NAFLD) with different stages of liver inflammation. Correlative analysis showed that changes in ^99m^Tc-cAbVCAM1–5 were strongly associated with established inflammation indicators, such as molecular biomarkers and histopathological inflammatory scores. Moreover, the imaging method was capable of perfectly detecting liver inflammation at any degree of severity, as reflected by area under the receiver operating characteristic (AUROC) values lying between 0.85 and 0.99. Such persuasive results provide a rationale for extending the ^99m^Tc-cAbVCAM1–5 investigation as a tool in the identification of liver inflammation in NAFLD patients, holding a pivotal medical need that is chronically neglected (172).

Scientists in a paper published in ATVB (2019) illustrated that vascular inflammation could be detected in real-time by binding ultrasound microbubbles with VCAM-specific Nbs (173). One group, taking this idea further, prepared a different imaging agent by producing technetium-labeled VCAM Nbs (^99m^Tc-cAbVCAM1-5). Besides mere diagnosis, the work of these modern imaging instruments is far-reaching. By enabling the exact detection of people with severely inflamed vasculature, they thus create opportunities for the impersonal administration of targeted drugs to the patients who will benefit the most (160, 174). Apolipoprotein-deficient (ApoE^−/−^) mice were used to study the *in vivo* behavior of AS lesions using cAbVCAM-1-5, an anti-VCAM-1 nanobody that was radio-labeled with fluorine-18 (^18^F). Using a necrotizing factor, the [^18^F]-FB-anti-VCAM-1 nanobody’s functionality and selectivity were examined through *in vitro* testing on the mouse endothelial cell line bEND5. [^18^F]-FB-cAbVCAM-1-5 (5 nM) only adheres to cells that are VCAM-1-positive, according to the gamma-counter results. In comparison to the control, mouse-detached aorta regions exhibited noticeably greater nanobody uptake at scores 1 (1.18 + 0.36%ID/g), 2 (1.49 + 0.37%ID/g), and 3 (1.61 + 0.41%ID/g). Labeled Nbs were found at the starting point of the ascending aorta and aortic arch, mainly in the segments with score 3, according to PET/CT images. Therefore, the [^18^F] FB-cAbVCAM-1–5 nanobody was used to precisely identify the lesions of AS that were visible in that area (175). In another study, researchers produced high-affinity Nbs against ICAM-1 that can block leukocyte adhesion or deliver protective agents directly to endothelial cells (176).

### 7.2 Nbs in diagnosing and treating ED

Once inflammation disrupts vascular integrity, restoring endothelial function requires targeted molecular intervention. Several studies have begun to explore this space. The two most promising candidates were investigated in the context of cardiac ischemia/reperfusion injury in a different study. Neutrophils’ attachment to an endothelial monolayer was decreased by nanobody-1 (Nb1) and Nb9. While Nb9 tended to impair isolated hearts’ performance, Nb1 did not affect left ventricular function *ex vivo*. Lastly, the mice’s survival rate was raised when Nb1 was applied *in vivo* at the start of reperfusion, but not Nb9 or a control Nb. Nonetheless, following treatment with Nb1, the infarct size was comparable to that following treatment with control Nb. To sum up, Nb1 effectively and selectively suppresses endothelial cell ATP release, which reduces leukocyte adhesion and enhances the end result of cardiac ischemia/reperfusion in mice. This calls for more research to determine the precise molecular mechanism behind Nb1’s advantageous effects (177). Therapeutic approaches have been developed as a result of mechanistic studies’ imaging results. Dabravolski and his colleagues demonstrated that endothelial pannexin-1 (Panx1) activation results in the release of mitochondrial DNA and ATP, which destabilizes plaques and causes vascular inflammation (178). Van Campenhout and his colleagues demonstrated that Panx1-blocking Nbs reduced endothelial inflammation by ATP blockade, which in turn decreased neutrophil adhesion and cytokine production. Since increased plaque formation was caused by persistent inflammatory signals within the disease process, the findings have direct relevance to atherosclerosis. When Panx1-blocking Nbs and anti-VCAM imaging probes are combined, researchers can develop a theranostic strategy that begins with lesion mapping and ends with the targeted delivery of antioxidant medications or Nbs to particular locations (85).

A blood glycoprotein called vWF is involved in both hemostasis and thrombosis. Acquired von Willebrand syndrome or excessive cleavage of the vWF multimers may be a consequence of elevated blood shear stress in cases of different cardiovascular diseases, e.g., congenital structural diseases, hypertrophic obstructive cardiomyopathy, or aortic stenosis (179). Raised levels of vWF have been termed one of the causes of aortic stenosis and are correlated with the risk of coronary heart disease and ischemic stroke (180). While searching for specific diagnostics, the llama-derived nanobody named AU/vWFa-11 seems to be a good alternative. SPR analysis showed that the nanobody binds the active A1 domain of vWF with the highest selectivity. After the nanobody has efficiently detected the protein in the laboratory-prepared solutions, the scientists have moved the experiment toward the detection of active vWF in human plasma (181). Subsequently, this feature helped to overcome a clinical challenge: prediction of bleeding risk and thrombocytopenia in von Willebrand disease type 2B patients. An immunosorbent assay with the AU/vWFa-11 nanobody was used to indirectly measure in plasma the specifically active form of vWF, i.e., the one that can bind to its platelet receptor, GPIb-α. In this method, nanobody-coated microtiter wells were incubated with diluted plasma samples; the bound vWF was then measured by anti-vWF polyclonal antibodies labeled with horseradish peroxidase (182). Furthermore, the nanobody AU/vWFa-11, just by itself, in its diagnostic capacity, helped to locate the exact mutations of the vWF A1 domain. Within the 24-member family scenario, the test distinguished 13 control members and 11 affected members bearing a mutation in exon 28 of the vWF gene. The AU/vWFa-11 nanobody selectively identified the mutated vWF monomers in the plasma of the affected individuals (183).

Another primary target for ED is GPCRs, and scientists have applied various nanobody-based methods to delve into their pharmacokinetics, anatomy, and expression alteration (165). To pinpoint a fresh strategy for regulating blood pressure, the scientists went through a library of synthetic camelid antibody fragments and eventually found Nbs that bind to the angiotensin II type 1 receptor (AT1R). One of the antagonist-mimicking nanobodies in a mouse model showed an antihypertensive effect equally effective as the drug losartan, a well-known origin (150). Furthermore, as therapeutic agents, these Nbs have been converted into an excellent vehicle to shed light on complicated biological structures. The study of the AT1R receptor structure was greatly facilitated by a nanobody named NbAT110, which directly led to understanding the bonding of activating peptides to the receptor—a process that was pretty much unknown due to the small size and high flexibility of the peptides. In addition to this, the work also revealed a lot of new information about the interaction surface, such as the peptide’s N-terminus being involved and a β-hairpin formed that slots into the partial β-barrel at the receptor’s own N-terminus (165).

In another field of medicine, nanobody technologies are unraveling the mysteries of a serious blood disorder. TTP is an extremely rare symptom cluster arising from the common cause of many small blood clots occurring in the body. The fundamental cause of the failure is a regulatory system malfunction involving vWF, which is a large protein entity. Normally, the protease ADAMTS13, by cleaving vWF, prevents the formation of ultra-large multimers. However, those suffering from TTP possess a deficiency in ADAMTS13—either congenital or acquired—hence the large multimers remain, triggering excessive platelet aggregation and consequently thrombi formation in small vessels leading to circular occlusions. Scientists identified a nanobody (anti-vWF Nb ALX-0681) that is capable of blocking spontaneous platelet aggregation and, at the same time, vWF activity in a baboon model (184). The first nanobody-derived approach to treating TTP, caplacizumab, was recently approved by the FDA and EMA. ALX-0081 is administered intravenously, while ALX-0681 is administered subcutaneously. Phase III clinical trial results demonstrated that ALX-0681 treatment results in decreased mortality, fewer recurrence cases, and faster disease resolution (162).

Although the field is still mostly preclinical, current data show that the use of nanobodies in cardiovascular disorders is progressively growing. The majority of research has been on creating molecular imaging probes, confirming disease-specific targets, and assessing proof-of-concept treatment approaches in experimental animals. Cardiovascular applications are currently constrained by the intricacy of vascular pathophysiology and the lack of clinically validated targets, in contrast to their well-established usage in oncology and inflammatory disorders. However, recent developments in molecular imaging and targeted delivery technologies, along with advances in nanobody engineering, have sped up development and created a solid basis for future translational research in cardiovascular therapy.

## 8 Role of Nbs in other CVDs

Anti-MMR Nbs were tested as a targeted therapy for AS, but the results were not very convincing. Apart from autoradiography and immunofluorescence, they also experimented with *in vivo* nuclear imaging SPECT/CT of technetium-99m (Tc-99m)-labeled Nb MMR3.49 in ApoE^−/−^ mice. The biodistribution of the Tc-99m-labeled anti-MMR Nb was quite positive in the spleen, liver, thymus, heart, lymph nodes, and bone marrow; nevertheless, the *in vivo* studies did not develop as expected, probably because the mouse model was used for the tests. Bala et al. (185) performed MMR suppression by the macrophage mannose receptor (MMR) using the nanobody cAbMMR3.49 as a tracer and visualized the process through SPECT/CT and PET/CT imaging methods. ^99m^Tc-labeled anti-MMR Nbs were injected into ApoE^−/−^ mice for SPECT/CT imaging, while ^18^F-labeled Nbs were given to Watanabe Heritable Hyperlipidemic (WHHL) rabbits for PET/CT imaging. Mice with ApoE^−/−^ and normal mice showed that MMR-positive tissues, such as lymph nodes, liver, and spleen, had a strong uptake of the ^99m^Tc-cAbMMR3.49 nanobody. Although the ApoE^−/−^ aorta was heavily absorbed by the AS, the number of Nbs was lower in AS with a higher score. According to the PET/CT scan, the nanobody was able to penetrate the aorta of both the rabbit model of atherosclerosis and the control rabbits. The attempts to visualize MMRs have failed, notwithstanding that the nanobody cAbMMR3.49 is specific for the MMR receptor (185).

Duy Tien Ta and his colleagues built a biosensor platform that can sense the VCAM-1 atherosclerosis biomarker by chemically modifying a nanobody and by chemically fusing it to azidified silicon wafers, boron-doped microcrystalline diamond films, and Biacore™ C1 sensor chips. To accomplish this, Nbs in this study were functionalized with an alkyne group at the C-terminus via the copper(I)-catalyzed Huisgen 1,3-dipolar azide-alkyne cycloaddition (CuAAC) to conjugate to an azide-functionalized substrate. The platforms that were developed helped Nbs to become more sensitive and to have a better binding capacity with the antigen, and the CuAAC method facilitated the orientation of Nbs’ surfaces with the substrate (186).

Numerous signal transduction pathways necessary for angiogenesis and cell migration depend on vascular endothelial growth factor receptor-2 (VEGFR-2). Behdani, along with colleagues, investigated the binding efficiency of different Nbs to the VEGFR-2 factor using SPR analysis. The same epitope is recognized by all tested Nbs; however, two Nbs, 3VGR19 and 4VR38, showed a higher affinity, with kD values ranging from 5.4 to 6.8 nM (187). An ELISA immunoassay can be used to clinically test for apolipoprotein-A1 (Apo-A1) in patients with cardiovascular disorders. A screen-printed carbon electrode (SPCE) coated with gold nanoparticles was used to create an immunosensor that utilized a similar idea. Following the binding affinity investigations, two Nbs were considered: Nb 11 as a capture Ab and Nb 19 strengthened by silver nanoparticle-loaded nano-hydroxyapatite (Ag-nHAP) as a detection Ab. For the detection of Apo-A1 at concentrations between 10−5 and 50 ng/mL, the calibration curve was created using linear sweep stripping voltammetry (LSSV) evaluation. Apolipoprotein-A1 was detected using an accurate and highly selective immunosensor with a detection limit of 0.02 pg/mL (188).

A traditional apolipoprotein electrochemical system A detection was looked into. It consists of three electrodes: an operational electrode made of glassy carbon enhanced with multi-walled carbon nanotubes and reduced graphene, a counter electrode made of platinum, and a reference electrode made of Ag/AgCl. Electrochemical spectroscopy (EIS) and sequential operando voltammetry were used to monitor the progression of the working electrode functionalization. After that, human Apo-A was probed using chronoamperometry at different concentrations (0.5–1,000 ng/mL), with a detection limit of 0.2 ng/mL. The biosensor’s selectivity was tested using human Apo-A, Apo-E, Apo-B, and 1 μg/mL of BSA, where only human Apo-A exhibited a more significant current response (189).

Light chain amyloidosis (AL) is the major source of heart failure, which is caused by the fibrillar deposition of immunoglobulin light chains (LCs) in the cardiac tissue. Nbs targeting these immunoglobulin fragments represent one of the newest strategies in fighting this deadly disease. The *in vitro* binding of the cardiotoxic light chain H3 to the nanobody is followed by in-depth studies such as microscale thermophoresis, isothermal titration calorimetry, multi-angle light scattering, and biolayer interferometry, which revealed that all the Nbs formed stable complexes with H3. The experiment with *Caenorhabditis elegans* suggested that the interaction inhibited the cardiotoxicity of H3 (190).

Nbs are engineered to directly associate with a single phosphorylation site on ryanodine receptor 2 (RyR2) to prevent the augmented Ca^2+^ leakage from the sarcoplasmic reticulum (SR). Phosphorylation is impeded at the site by Nbs specific for RyR2 *in vitro*, as well as *in vivo* studies. The report claims that the improvement of systolic and diastolic dysfunction has been achieved through the regulation of the AR185 protein expression, and hence, the delivery of the AR185 gene hampers the release of SR calcium in the infected cardiomyocytes. Cardiomyocytes with the fluorescent probe incubated in the presence of calcium ions bound to Fluo-5N were recorded and analyzed by laser scanning confocal microscopy. Therefore, the presented data support intracellular Nbs therapy as a potential tool for rat heart disease intervention; however, the current available evidence is still insufficient for its proposal to human patients (191).

CVD is associated with altered expression of plasminogen activator inhibitor-1 (PAI-1). PAI-1 is a member of the serine protease inhibitor family, the major physiological role of which is to inhibit tissue-type plasminogen activators and urokinase. Numerous molecules, including peptides and antibodies, have been created to act as PAI-1 inhibitors; however, the conformational plasticity of these molecules significantly hampers their functionality. Additionally, several PAI-1-interacting antibody and nanobody libraries cited in the literature have been disclosed here as well (192). The Nb93 nanobody can bind to the reactive center loop (RCL) on the surface of PAI-1, thereby blocking the PA binding site of the molecule. According to a mutagenesis study, the epitope that the nanobody is targeting is close to the RCL and includes the amino acids Glu244, Glu242, Lys243, and Glu350 (193).

An extremely dangerous condition, obesity is typically linked to metabolic syndrome and several illnesses, including IDDM type 2, CVD, and hypertension. Despite the fact that few therapies have been created and recognized, some were discontinued due to their numerous side effects. Researchers used an intracellular nanobody to study the receptor GPR75 (G protein receptor), a promising prospect for the treatment of obesity, in an effort to tackle the lack of viable approaches. They studied the GPR75–nanobody complex (NbH3) using cryo-electron microscopy (Cryo-EM) and gathered data on receptor stimulation, which is critical data in the hunt for a novel anti-obesity therapeutic agent (194).

Research indicates that anomalies in Ca^2+^ signaling, which result in changes to the contraction and relaxation states, are the primary cause of heart failure. The sarco-endoplasmic reticulum Ca^2+^-ATPase (SERCA2a) is downregulated in this condition due to changes in its activity and increased levels of non-phosphorylated phospholamban. Researchers have discovered VHH Nbs that are capable of binding to phospholamban in different conformational states with incredibly high specificity and changing its function, making phospholamban an appealing target for new approaches to therapy. They showed that this nanobody can increase cardiomyocyte activity in a murine model by using an adenovirus to transport it (195).

When considered collectively, the studies that are now available indicate that the most valuable use of nanobodies may not be to replace current treatments but rather to enhance them by extremely specific targeting, early illness detection, and precise drug delivery. The evidence is still fragmented, with most studies concentrating on individual targets rather than integrated disease pathways, despite encouraging findings being published across several cardiovascular disorders. Therefore, in order to properly characterize the therapeutic role of nanobodies in cardiovascular illness, future research should go beyond proof-of-concept studies and give priority to comparative evaluations, delivery strategy optimization, and validation in clinically relevant models.

## 9 Benefits and limitations

The most significant benefits of Nbs over antibodies include their capacity to reach difficult-to-reach locations, their ability to bind to various target molecule regions, their high specificity and extremely low sensitivity, the fact that they have no side effects because they are quickly removed from the body, and, of course, their ease of production and low cost. Within 3 h after injection, researchers were able to use single-photon emission computed tomography imaging with ^99m^Tc-labeled Nbs due to the short blood half-life. Some of the most significant biophysical properties of Nbs in relation to CVD applications in the domains of diagnosis and treatment are listed in **Table 5**.

**Table 5:** Biophysical properties of Nbs in the diagnosis and therapy of cardiovascular diseases (CVDs).

Biophysical property	Benefits	Drawbacks	Applications	References
Strong antigen-binding affinity	Exhibits high specificity for diverse receptors and biomarkers, supporting selective targeting of disease mediators	Suboptimal design or mutation may cause non-specific interactions or reduced selectivity	Structural mapping of cardiovascular receptors; inhibition of inflammatory and atherogenic pathways	Jovčevska et al. (162)
Low immunogenic potential	Generally well tolerated, with a low tendency to trigger immune responses, allowing for repeated administration	Still requires humanization in some therapeutic settings to minimize the risk of anti-drug antibody development	*In vivo* cardiovascular imaging and long-term therapeutic delivery	Zhu et al. (196)
Compact molecular size	Small molecular mass enables easy diffusion through tissues and access to recessed or dense vascular regions	Rapid renal clearance due to filtration below the glomerular threshold limits circulation time	Targeted molecular imaging and receptor-based nanobody therapies	Jovčevska et al. (162)
Single-domain structure	Provides high affinity and structural stability while minimizing cross-reactivity; ideal for precision targeting	Reduced efficiency in binding to small ligands such as peptides or haptens	Biosensor and immunosensor platforms; targeted drug delivery systems	Yu et al. (197)
Thermal and chemical stability (extended CDR3 loops)	Capable of refolding after denaturation; retains biological function even under extreme thermal or chemical stress	Can occasionally undergo unwanted intermolecular interactions or aggregation	Imaging of vascular inflammation and monitoring of plaque progression	Yu et al. (197)
High solubility in aqueous media	Excellent solubility enables easy preparation of injectable formulations and functional conjugates	–	Diagnostic assays include ELISA, electrochemical biosensors, and imaging conjugates	Zhu et al. (196)

Numerous diagnostic instruments based on Nbs have been created thus far, with outstanding sensitivity at the nano- and picomolar levels and proven specificity to the intended target. However, despite the potential benefits of these methods for CVD prevention and early diagnosis, they still need a lot of work to validate *in vivo* models, and they frequently run into ethical problems that slow down the process. Some problems associated with sophisticated imaging techniques that use Nbs include low adherence efficiency, inadequate selectivity, and differentiation from the background signal in the desired region (173, 185).

Using various methods, Nbs have proven effective in a variety of applications, such as helping to deliver medications to a particular target, activating or blocking a particular biomarker, or acting as labeling molecules. Numerous researchers have demonstrated that Nbs seem to be adaptable. As previously demonstrated, they can even function as potent inhibitors or identify the structural properties of desired molecules. Individuals with thrombotic thrombocytopenic purpura have already benefited from the successful application of nanobody technology in the pursuit of novel and effective CVD therapies, and the introduction of caplacizumab into the market may mark the beginning of an exciting new era for the therapy of CVD patients. However, there are worries about the safety of Nbs as therapeutic agents because the human body is unable to produce them, mainly because of the potential for them to cause undesirable immune reactions. Scientists have proposed surface modification of specific regions with human sequences to prevent the possible adverse effects, but this could result in low solubility (198, 199).

Nanobody-based molecular imaging probes have the potential to revolutionize clinical medicine by combining advances in imaging technology with a better understanding of the biology of disease. Molecular imaging provides a cost-effective and sensitive way to identify atheromas, which are likely to cause thrombotic issues (200). Furthermore, research has shown that information about the physiological state of atherosclerosis-affected cells can be gathered by combining PET with Nbs that target various receptors.

## 10 Future perspectives

Future research on vascular inflammation and ED and CVD treatment will concentrate on enhancing nanobody therapeutic stability, specificity, and their ability to translate to human use. Nbs show great promise for targeted vascular inflammation treatment because they bind strongly to oxidative and inflammatory targets, which damage endothelial cells, including VCAM-1, ICAM-1, and TNF-α. The therapeutic use of Nbs faces ongoing challenges because they are quickly removed from the body through the kidneys, have short durations in the bloodstream, and may trigger immune responses. Scientists now focus on developing PEGylated Nbs and nanobody–Fc fusion constructs to enhance their pharmacokinetic properties and extend their circulation duration to overcome these obstacles. The development of bispecific and multivalent Nbs, which enable simultaneous targeting of multiple endothelial repair and oxidative stress regulation pathways, will enhance their anti-inflammatory effects. The combination of nanobody-based imaging with drug delivery systems (theranostics) provides promising capabilities for real-time vascular injury assessment and targeted therapeutic delivery. The implementation of these strategies shows potential to enhance endothelial protection and vascular function, but their translation to clinical practice depends on successful large-scale validation and production system optimization.

## 11 Conclusion

Because of their tiny size, high specificity, and adaptable engineering capabilities, nanobody-based approaches hold great promise for improving the detection and treatment of endothelial dysfunction and vascular inflammation. Promising vascular targets such as VCAM-1, ICAM-1, VEGFR2, and pannexin-1 (Panx1) have been discovered recently, underscoring the growing relevance of nanobodies in molecular imaging and tailored treatment methods. The use of nanobodies in clinical cardiology treatment is still in its infancy despite these positive advancements. The majority of the information that is now available comes from preclinical research, and before widespread therapeutic application can be attained, a number of issues must be resolved, including quick renal clearance, a brief circulation half-life, distribution strategy optimization, and a lack of clinical validation. Some of these restrictions should be addressed by further developments in nanobody engineering and a deeper comprehension of endothelial biology. To completely determine the safety and therapeutic potential of nanobodies in cardiovascular illness, future research should concentrate on verifying novel vascular targets, enhancing pharmacokinetic characteristics, and carrying out carefully planned clinical investigations.
